# A Peptide Derived from Phosphoinositide 3-kinase Inhibits Endocytosis and Influenza Virus Infection

**DOI:** 10.1247/csf.19001

**Published:** 2019-03-21

**Authors:** Yoichiro Fujioka, Aya O. Satoh, Kosui Horiuchi, Mari Fujioka, Kaori Tsutsumi, Junko Sasaki, Prabha Nepal, Sayaka Kashiwagi, Sarad Paudel, Shinya Nishide, Asuka Nanbo, Takehiko Sasaki, Yusuke Ohba

**Affiliations:** 1 Department of Cell Physiology, Faculty of Medicine and Graduate School of Medicine, Hokkaido University, N15W7, Kita-ku, Sapporo 060-8638, Japan; 2 Department of Biological Sciences and Engineering, Faculty of Health Sciences, Hokkaido University, N12W5 Kita-ku, Sapporo 060-0812, Japan; 3 Department of Biochemical Pathophysiology, Medical Research Institute, Tokyo Medical and Dental University, 1-5-45 Yushima, Bunkyo-ku, Tokyo 113-8510, Japan; 4 Whitman Center, Marine Biological Laboratory, 7 MBL Street Woods Hole, MA 02543, USA

**Keywords:** signal transduction, endocytosis, endosome, imaging, influenza virus

## Abstract

Endocytosis mediates the internalization and ingestion of a variety of endogenous or exogenous substances, including virus particles, under the control of intracellular signaling pathways. We have previously reported that the complex formed between the small GTPase Ras and phosphoinositide 3-kinase (PI3K) translocates from the plasma membrane to endosomes, signaling from which thereby regulates clathrin-independent endocytosis, endosome maturation, influenza virus internalization, and infection. However, the molecular mechanism by which the Ras-PI3K complex is recruited to endosomes remains unclear. Here, we have identified the amino acid sequence responsible for endosomal localization of the Ras-PI3K complex. PI3K lacking this sequence failed to translocate to endosomes, and expression of the peptide comprising this PI3K-derived sequence inhibited clathrin-independent endocytosis, influenza virus internalization, and infection. Moreover, treatment of cells with this peptide in an arginine-rich, cell-penetrating form successfully suppressed influenza virus infection in vitro and ex vivo, making this peptide a potential therapeutic agent against influenza virus infection.

## Introduction

Endocytosis is a cellular mechanism by which a variety of substances are engulfed and transported from outside a cell to the inside. Several taxonomies are available to characterize endocytosis. For instance, endocytosis can be roughly divided into pinocytosis (cell drinking) and phagocytosis (cell eating) based on the type of substance incorporated and their mechanisms. Endocytosis pathways can also be subdivided into four categories, including receptor-mediated (or clathrin-mediated), caveolae-dependent, clathrin-independent (including macropinocytosis and micropinocytosis), and phagocytosis, based on the regulatory mechanism utilized, such as coated pits lined with proteins (Godlee and Kaksonen, 2013; Maldonado-Báez *et al.*, 2013; McMahon and Boucrot, 2011; Merrifield and Kaksonen, 2014). Endocytosis is a mandatory process in which cells uptake important chemical substances, as large polar molecules (such as proteins) cannot penetrate the aliphatic plasma membrane in a passive manner. Low-density lipoproteins, transferrin, growth factors, antibodies, and many other ligands can be internalized into cells by binding their specific receptors and then undergoing the subsequent coated pit formation and clathrin-mediated endocytosis (Marsh and McMahon, 1999).

Pathogens are also internalized into cells via endocytosis. In antigen-presenting cells, bacteria or other pathogenic microorganisms are internalized via phagocytosis, digested, and degraded in lysosomes, the products of which are then presented at the cell surface for recognition by helper T cells. *Shigella*, *Salmonella enterica*, and *Mycobacterium tuberculosis* enter cells via endocytosis to reach a milieu in which they can proliferate. In addition to bacteria, both non-enveloped viruses (e.g., *Adenoviridae*) and enveloped viruses (e.g., influenza viruses) are internalized into cells via endocytosis, after which they release their genomes into the cytoplasm for replication.

In addition to extracellular and exogenous substances, membrane proteins are also internalized into cells via endocytosis, which eventually contributes to the regulation of signal transduction pathways. For instance, endocytosis promotes the translocation of membrane receptors from the plasma membrane to endosomes, resulting in a decreased probability of receptors associating with ligands by sequestering receptors from the cell surface. This thereby results in desensitization to the ligands and signaling downregulation. Moreover, endocytosis regulates the fate of signaling molecules via endosomal sorting (i.e., recycling versus degradation). Endosomes are also attracting attention not only as organelles that carry many substances but also as platforms that positively emit signals themselves. For example, nerve growth factor receptors are known to activate different downstream factors depending on their localization; activation at the cell membrane and in endosomes is responsible for cell proliferation and differentiation, respectively (Grimes *et al.*, 1996; Murphy *et al.*, 2009). On the other hand, endocytosis machinery is regulated by a variety of signaling pathways, including phosphoinositide 3-kinase (PI3K) signaling.

The lipid kinase PI3K phosphorylates phosphoinositides at the 3' position of the inositol ring (Whitman *et al.*, 1988) and plays roles in a variety of cellular functions, including cell growth, proliferation, differentiation, motility, survival, and intracellular membrane trafficking, through the recruitment of pleckstrin homology (PH) domain-containing proteins (Vanhaesebroeck *et al.*, 2012). Activation of PI3K is promoted by at least four independent pathways. First, the p110β catalytic subunit of PI3K can be activated by G protein-coupled receptors (Katso *et al.*, 2001). The other three pathways are initiated by the activation of receptor tyrosine kinases (RTKs) via ligand binding. The Src homology 2 (SH2) domain in the regulatory subunit p85 of PI3K is directly recruited to phosphorylated tyrosines (YXXM) within RTKs (Domchek *et al.*, 1992), triggering the catalytic activation of PI3K. p85 can also be recruited to another phosphorylated tyrosine-containing motif, YXN, via adaptor proteins, including growth factor receptor-bound protein 2 (Grb2) and Grb2-associated binder 1 (GAB1) (Pawson, 2004). Finally, the fourth way to activate PI3K is via Ras. Recruitment of Grb2, some fraction of which constitutively binds the Ras activator son of sevenless (Sos), results in activation of Ras, which activates p110 independently of p85 by the binding of Ras to the Ras-binding domain (RBD) of p110 (Ong *et al.*, 2001).

We have previously reported that the Ras-PI3K complex is translocated to endosomes, and calcium-dependent activation of Ras-PI3K signaling are required for the regulation of clathrin-independent endocytosis, endosomal maturation, influenza virus internalization, and infection (Fujioka *et al.*, 2018, 2011, 2013; Tsutsumi *et al.*, 2009). However, the molecular mechanism by which the Ras-PI3K complex is recruited to the endosome remains unclear. Here, we have identified the amino acid sequence that is crucial for endosomal localization of the Ras-PI3K complex, and we named this sequence RAPEL (Ras-PI3K endosomal localization). The forced expression of this peptide inhibited endocytosis, influenza virus internalization, and infection without affecting canonical Ras or PI3K-related signaling. Given that its introduction in peptide form was also effective in inhibiting influenza virus infection, we offer a novel peptide-based therapy targeting influenza virus infection and other endocytosis-related diseases.

## Materials and Methods

### Sequence analysis

The amino acid sequences of PI3K p110α (NP_006209.2), PIK3 p110β (NP_006210.1), p110γ (NP_001269356.1), p110δ (NP_005017.3), c-Raf (NP_002871.1), B-Raf (NP_004324.2), A-Raf (NP_001645.1), RalGDS (NP_006257.1), Rgl1 (NP_055964.3), Rgl2 (NP_004752.1), Rgl3 (NP_001155088.2), and Rlf (AAC52724.1) were obtained from the NCBI Protein Database. From these sequences, the RBDs were identified with the use of the CD Search tool of the NCBI Conserved Domain Database. Multiple sequence alignment was performed with the use of ClustalW.

### Reagents and antibodies

Recombinant human EGF was purchased from PeproTech (Rocky Hill, NJ, USA). Hoechst 33342, Alexa Fluor 546-labeled dextran (10 kDa), rhodamine B-labeled dextran (70 kDa), Alexa Fluor 546-labeled transferrin, and Alexa Fluor 594- and Alexa Fluor 647-conjugated antibodies against mouse, rabbit, and rat immunoglobulin G were obtained from Thermo Fisher Scientific (Carlsbad, CA, USA). Vps34-IN1 was purchased from Funakoshi (Tokyo, Japan). Horseradish peroxidase-conjugated sheep antibodies against mouse, donkey, and rabbit immunoglobulin G were purchased from GE Healthcare (Little Chalfont, UK), and horseradish peroxidase-conjugated donkey antibodies against goat immunoglobulin G were purchased from Promega (Madison, WI, USA). Antibodies against EEA-1 (610456) were obtained from BD Biosciences (San Jose, CA, USA). Antibodies against GST (5A7) were purchased from Wako Pure Chemical Industries, Ltd. (Osaka, Japan). Antibodies against ERK (9102S), phosphor-ERK (9101S), Akt (9272S), phosphor-Akt (9271S), and Rab7 (9367S) were purchased from Cell Signaling Technology (Danvers, MA). Antibodies against GFP (598), HA (3F10), NP (ab20343), β-actin (sc-47778), and Ras (Ab-3) were obtained from Medical & Biological Laboratories (MBL; Nagoya, Japan), Roche (Indianapolis, IN, USA), Abcam (Cambridge, UK), Santa Cruz Biotechnology (Santa Cruz, CA), and Oncogene (Cambridge, MA, USA), respectively.

### Plasmids

pCAGGS-VN-H-Ras WT and G12V, pCXN2-Flag-p110γ RBD-VC, pCXN2-Flag-H-Ras WT, pCAGGS-EGFP-H-Ras G12V, pCAGGS-Venus-H-Ras G12V, pCXN2-3×HA-p110γ, pCAGGS-ECFP-Rab5, and pRaichu-Ras 146X were described previously (Fujioka *et al.*, 2011; Mochizuki *et al.*, 2001; Ohba *et al.*, 2003; Tsutsumi *et al.*, 2009). pCMV-TagRFP-EEA-1 was purchased from Addgene (Cambridge, MA, USA). cDNA for p110γ RBD ΔRAPEL was generated by PCR with the following primers: ΔRAPEL_F (5'-CCCTCGAGCAGACCATTAAGGTCTCACC-3') and RBD_R (5'-GGGCGGCCGCCGTCTGGAGGCGTGTCCAGT-3').

These sequences were then subcloned into the *Xho*I/*Not*I sites of pCXN2-Flag-p110γ RBD-VC to obtain pCXN2-Flag-p110γ RBD ΔRAPEL-VC. The coding sequences of wild-type p110γ RBD and p110γ RBD ΔRAPEL were also subcloned into the *Xho*I/*Not*I sites of the pCAGGS-ECFP, pCAGGS-mRFP, or pCAGGS-GST vectors to obtain pCAGGS-CFP-p110γ RBD, pCAGGS-mRFP-p110γ RBD, or pCAGGS-GST-p110γ RBD, and pCAGGS-ECFP-p110γ RBD ΔRAPEL, pCAGGS-mRFP-p110γ RBD ΔRAPEL, or pCAGGS-GST- p110γ RBD ΔRAPEL, respectively.

The coding sequences of RAPEL and its derivatives (CT9, CT20, NT8, and M11) were amplified by PCR with the following primers: CT9_F (5'-GGCTCGAGATCGTCATTCACCGCAGC-3') and CAGR2 (5'-AGATGCTCAAGGGGCTTCATGATG-3'), CT20_F (5'-GGCTCGAGCTGTGGAAGAAGATTGCC-3') and CAGR2, CFP_F and NT8_R (5'-TAGCGGCCGCTCAGTACTCCGGGAGGGGC-3'), and CFP_F and NT19_R (5'-TAGCGGCCGCTCAGAAGATGCAGTTGTTGGC-3'). These sequences were then subcloned into the *Xho*I/*Bgl*II or *Eco*RI*/Not*I sites of pCAGGS-ECFP to obtain pCAGGS-ECFP-RAPELs. To obtain the mutant RAPEL-M11 in which one or two lysine residues were substituted by glutamate (EE, EK, or KE), the oligonucleotide pairs EE_F (5'-TCGAGCTGTGGGAGGAGATTGCCAACAACTGCATCTTCTGAGC-3') and EE_R (5'-GGCCGCTCAGAAGATGCAGTTGTTGGCAATCTCCTCCCACAGC-3'), EK_F (5'-TCGAGCTGTGGGAGAAGATTGCCAACAACTGCATCTTCTGAGC-3') and EK_R (5'-GGCCGCTCAGAAGATGCAGTTGTTGGCAATCTTCTCCCACAGC-3'), or KE_F (5'-TCGAGCTGTGGAAGGAGATTGCCAACAACTGCATCTTCTGAGC-3') and KE_R (5'-GGCCGCTCAGAAGATGCAGTTGTTGGCAATCTCCTTCCACAGC-3') were respectively annealed and then subcloned into the *Xho*I/*Not*I sites of pCAGGS-ECFP.

The cDNAs encoding full-length p110γ that harbor the above mutations were obtained by PCR-based mutagenesis with the following primers and pBlueScript II SK(+)-p110γ (Fujioka *et al.*, 2011) as a template: p110γEE_F (5'-CGGAGTACCTGTGGGAGGAGATTGCCAACAACTGC) and p110γEE_R (5'-GCAGTTGTTGGCAATCTCCTCCCACAGGTACTCCG), p110γEK_F (5'-CGGAGTACCTGTGGGAGAAGATTGCCAACAACTGC) and p110γEK_R (5'-GCAGTTGTTGGCAATCTTCTCCCACAGGTACTCCG), p110γKE_F (5'-CGGAGTACCTGTGGAAGGAGATTGCCAACAACTGC) and p110γKE_R (5'-GCAGTTGTTGGCAATCTCCTTCCACAGGTACTCCG). After eliminating the template DNA by *Dpn*I digestion, the resulting PCR products were then self-ligated and introduced into *E. coli* JM109 competent cells. The coding sequence of p110γ mutants were cleaved out by *Xho*I/*Not*I digestion and subcloned into the *Xho*I/*Not*I sites of pCXN2-3×HA-p110γ. cDNA for RAPEL-deleted-p110γ was generated by PCR with the primers ΔRAPEL_F and PI3K_R (5'-CCGGGCCCTCAGCTAGTTAGCGGCCGCCGGCTGAATGTTTCTCTCC-3') and subcloned into the *Xho*I*/Not*I sites of pCXN2-3×HA-p110γ. All the PCR products were confirmed by sequencing analysis.

### Cell culture

HEK293T (CRL-11268), MDCK (CCL-34), BEAS-2B (CRL-9609), and A549 (CCL-185) cells were obtained from American Type Culture Collection (Manassas, VA, USA), and Cos-1 cells (JCRB9082) were from purchased from the Japan Collection of Research Bioresources Cell Bank of the National Institutes of Biomedical Innovation, Health and Nutrition (Ibaraki, Osaka, Japan). These cells were maintained under a 5% CO_2_ humidified atmosphere at 37°C in Dulbecco’s Modified Eagle’s Medium (DMEM, Sigma-Aldrich) supplemented with 10% fetal bovine serum (FBS, Thermo Fisher Scientific). Expression vectors were introduced into cells by transfection with the use of Polyethylenimine “Max” (Polysciences, Warrington, PA, USA). Mouse embryonic fibroblasts deficient in *PIK3CG* were prepared as described (Sasaki *et al.*, 2000), and maintained in DMEM supplemented with 10% FBS. Expression vectors for wild-type and mutant PI3Kγ were linearized by *Sca*I and introduced into the knockout cells using nucleofection according to the manufacturer’s recommendations (Amaxa Biosystems, Cologne, Germany). Starting at 2 days, the cells were cultured in DMEM containing 0.5 mg/ml G418 sulfate (Sigma), and the resistant colonies were collectively isolated. Expression of each protein was confirmed by immunoblotting (see Fig. S5B). Stably transfected cells were maintained in DMEM supplemented with 10% FBS and 0.2 mg/ml G418. The absence of mycoplasma contamination was confirmed using a PCR Mycoplasma Test Kit (Takara, Shiga, Japan).

### Virus preparation and plaque assay

MDCK cells were infected with IAV strains A/Puerto Rico/8/34 (H1N1; PR8) or A/Aichi/2/68 (H3N2; Aichi) at a multiplicity of infection (MOI) of 0.0001 plaque-forming units (PFUs) per cell for 48 hours at 35°C. Viruses isolated by high-speed centrifugation were resuspended in phosphate-buffered saline (PBS) and stored at –80°C until use. The amount of infectious virus in the virus solution or culture media was determined with a plaque assay (Pleschka *et al.*, 2001). In brief, confluent MDCK cell monolayers in 12-well plates were inoculated with conditioned medium of virus-infected cells. After 1 hour of culturing, the supernatants were removed, and the cells were overlaid with minimal essential medium containing 1% Bacto-agar (Sigma-Aldrich) and trypsin (5 mg/ml, Sigma-Aldrich). Plaques were counted after incubation at 35°C under 5% CO_2_ for 2 days.

### Peptides

The peptides used in this study were chemically synthesized using (9-fluorenylmethyloxycarbonyl)-solid phase peptide synthesis. Fluorescent labeling of the peptides was conducted by treatment with 5-maleimidofluorescein isothiocyanate in dimethylformamide methanol followed by reverse phase high-pressure liquid chromatography purification (purity >90%). The fidelity of the products was ascertained by time-of-flight mass spectrometry. Peptide synthesis and purification were performed by BEX (Tokyo, Japan). For peptide delivery, 1×10^5^ MDCK or 293T cells were seeded in a collagen-coated, 35-mm-diameter glass-base dish (Asahi Techno Glass Co, Yoshida, Shizuoka, Japan) and grown to 60–70% confluency. Each well was washed three times with PBS and then incubated with 100 μM peptide in serum-free DMEM for 1 hour at 37°C. Cells were then washed three times with PBS, incubated for 24 hours, and subjected to analyses.

### Wide-field fluorescence microscopy

Cells were imaged with an IX-83 microscope (Olympus, Tokyo, Japan) equipped with a BioPoint MAC6000 filter and shutter control unit (Ludl Electronic Products, Hawthorne, NY, USA), an automated XY-stage (Chuo Precision Industrial, Tokyo, Japan), an sDISK spinning disk confocal microscope (Andor Technology, Belfast, UK), and a Rolera EM-C^2^ electron multiplying cooled charge-coupled device camera (QImaging, Surrey, BC, Canada). For epifluorescence microscopy, cells were illuminated with a SOLA Light Engine (Lumencor, Beaverton, OR, USA) and the following excitation and emission filters were used: FF02-438/24 and FF01-483/32 (Semrock, Rochester, NY, USA) for CFP; BP470-490 and BP510-550 (Olympus) for FITC; FF02-438/24 and FF01-542/27 for FRET; and BP520-550 and BA580IF (Olympus) for Alexa Fluor 594 and mRFP. For spinning-disk confocal imaging, cells were illuminated with a four-line (405, 488, 561, and 633 nm) laser module (Spectral Applied Research, Hamilton, ON, Canada) and the following emission filters were used: FF01-483/32 for CFP, FF01-542/27 for FRET, and BA580IF for TagRFP. Typical exposure times for 2×2 binning were 0.3 s. MetaMorph software (Universal Imaging, West Chester, PA, USA) was used to control the electron multiplying charge-coupled device camera, confocal unit, stage, illumination, and filter wheels as well as for cell imaging data analysis.

Imaging of BiFC and FRET, and their data analysis were performed essentially as described previously (Fujioka *et al.*, 2013; Ohba *et al.*, 2003; Tsutsumi *et al.*, 2009). In brief, cells plated on a collagen-coated, 35-mm-diameter glass-base dish were transfected with expression vectors. After 24 hours, cells were serum-starved in phenol red-free DMEM/F12 (Thermo Fisher Scientific) for 4 hours and placed in a stage-top incubation chamber (Live Cell Instrument, Soul, Korea) that was maintained at 37°C. Beginning at 10 min, cells were stimulated with 100 ng/ml EGF. BiFC images were acquired using a spinning disk mode, whereas FRET images were obtained using epifluorescence mode. To represent FRET efficacy, the FRET/CFP emission ratio was calculated as the quotient of background-subtracted FRET and CFP images. To stabilize jittery image stacks of time-lapse data set, ‘Image Stabilizer’ plug-in of ImageJ (https://imagej.nih.gov/ij) was used. MetaMorph software converts image stacks of time-lapse data set into one QuickTime movie.

### Immunofluorescence

For immunofluorescence staining, cells were fixed with 3% paraformaldehyde for 15 min at room temperature, permeabilized with 0.1% Triton X-100 in PBS for 4 min at room temperature and then incubated with 1% bovine serum albumin to block nonspecific antibody binding. The cells were further incubated with primary antibodies (NP, 1:1,000; c-Myc, 1:1,000; HA, 1:1,000; EEA-1, 1:1,000) overnight at 4°C, after which immune complexes were detected by incubation for 1 hour at room temperature in the dark with Alexa Fluor 594- or Alexa Fluor 647-conjugated secondary antibodies (1:250). Images were acquired with the wide-field fluorescence microscope (infection assay in 2D culture) or an FV10i confocal microscope (other experiments, Olympus).

### Quantification of endosomal localization

For analysis of the endosomal localization of the Ras-PI3K complexes, confocal images were acquired from cells expressing VN-Ras, PI3K RBD-VC, and TagRFP-EEA-1 and subjected to background subtraction. Vesicles positive for a BiFC signal (from Ras-PI3K complex) or TagRFP (EEA-1) were extracted with the use of the ‘Top Hat’ module of MetaMorph software, and colocalization of the Ras-PI3K complex with EEA-1 was quantified with the use of the ‘measure colocalization’ function of the software.

### Endocytosis

For detection of clathrin-independent and clathrin-mediated endocytosis, cells plated on collagen-coated glass-bottom dishes (35-mm diameter) were incubated with Alexa Fluor-conjugated dextran (500 μg/ml) for 10 min or transferrin (500 μg/ml) for 30 min at 37°C. After incubation, cells were washed twice with acid wash buffer [0.2 M glycine, 150 mM NaCl (pH3.0)] followed by additional washing three times with PBS to remove non-internalized substances (Kameyama *et al.*, 2007). Images were acquired with a wide-field fluorescence microscope (IX-83) or a confocal microscope (FV10i) and subjected to background subtraction. Total fluorescence intensity within the cells was quantitated with MetaMorph software.

### Immunofluorescence-based influenza virus internalization and infection assay

Immunofluorescence-based virus internalization and infection assays were performed as described previously (Fujioka *et al.*, 2013, 2018). In brief, serum-deprived cells were incubated with IAV at an MOI of 10 PFU per cell for 1 hour (internalization assay) or at an MOI of 1 PFU per cell for 4 hours (infection assay). Cells were washed with acidic buffer, fixed with 3% paraformaldehyde for 15 min at room temperature, and then subjected to immunofluorescence with the use of an anti-NP antibody (1:1,000) and an anti-Rab7 antibody (1:500). Nuclei were visualized by staining with Hoechst 33342. Images were acquired with the FV10i confocal microscope for the internalization assay and for the infection assay in a 3D culture system or with the wide-field epifluorescence microscope for the infection assay in 2D cultures.

To quantify virus internalization, confocal sections were acquired from the top to the bottom of the cells. Vesicles positive for NP or Rab7 were extracted with the use of the ‘granularity’ module of MetaMorph software, and the colocalization of NP and Rab7 was evaluated with the use of the ‘measure colocalization’ function. For analysis of virus infection, a region of each individual cell was identified by the “watershed” algorism based on the nuclear staining and total fluorescence intensities of NP were gauged within the region.

### Immunoblotting

Unless otherwise specified, cells were lysed in RIPA buffer [50 mM Tris-HCl (pH 7.4), 150 mM NaCl, 1 mM EDTA, 1% Nonidet P-40, 0.1% SDS, 0.5% sodium deoxycholate, 1 mM Na_3_VO_4_] for 30 min on ice. The lysates were centrifuged at 20,000 *g* for 10 min at 4°C, and the resulting supernatants were subjected to SDS-PAGE. The separated proteins were transferred to polyvinylidene difluoride membranes (Bio-Rad, Hercules, CA, USA) and subjected to immunoblot analysis. Immune complexes were detected with the use of ECL Western Blotting Detection Reagent (GE Healthcare) and an LAS-1000UVmini image analyzer (Fujifilm, Tokyo, Japan).

### Pull-down assays

Cells were lysed in lysis buffer [50 mM Tris-HCl (pH 7.4), 150 mM NaCl, 5 mM MgCl_2_, 1% Nonidet P-40, 0.5% sodium deoxycholate, 0.1% SDS, 1 mM Na_3_VO_4_]. After centrifugation, the resulting supernatants were incubated for 60 min at 4°C with glutathione Sepharose beads (GE Healthcare), washed three times with lysis buffer, eluted in SDS sample buffer, and subjected to immunoblot analysis.

### 3D culture

BEAS-2B cells were cultured in Matrigel (CORNING, Corning, NY, USA) in a 35-mm-glass-base dish for 5 days. After confirming monolayer formation, the cells were further cultured in the presence or absence of the synthetic peptides for 1 hour, and after washing, further incubated for 24 hours. The cells were then infected with PR8 at an MOI of 1 PFU per cell for 4 hours and subjected to an immunofluorescence-based infection assay with the use of antibodies to IAV NP. Nuclei were counterstained with Hoechst 33342. The samples were observed under the confocal microscope FV10i.

### Statistical analysis

For data presented as box-and-whisker plots, the highest and lowest boundaries of the box represent the 25th and 75th percentiles, respectively, the whiskers above and below the box designate the 5th and 95th percentiles, respectively, and the line and dot within the box indicate the median and mean, respectively. Other data are presented as the mean±s.e.m. Such data were obtained from at least three independent experiments (unless indicated otherwise) and were compared by Student’s *t*-test (parametric test between two conditions), Welch’s *t*-test (nonparametric test between two conditions) or one-way analysis of variance (ANOVA) followed by post-hoc Tukey honestly significant difference (HSD) test (among multiple conditions). Time series data sets were compared by repeated-measures ANOVA (one condition), multivariate analysis of variance (MANOVA) (between two conditions), or MANOVA with Bonferroni correction (among multiple conditions). No statistical methods were used to predetermine sample size. Studies were performed unblinded.

## Results

### Identification of the amino acid sequence required for endosomal localization of the Ras-PI3K complex

**Fig. 1:**
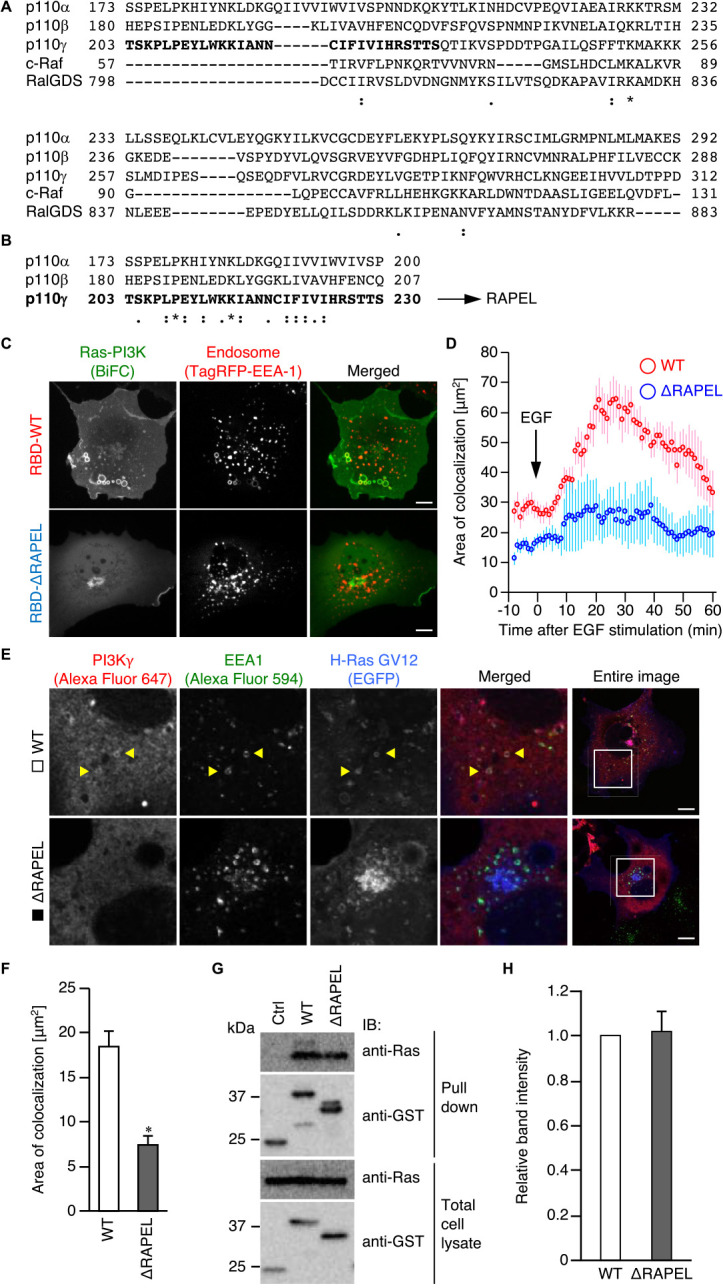
Deletion of RAPEL in PI3K RBD resulted in the inability of the Ras-PI3K complex to undergo endosomal localization. (A, B) Multiple alignments of amino acid sequences of the RBDs of Ras effectors (A) and of 28 N-terminal amino acids of PI3K RBDs (B). “.”, “:”, and “*” indicate low homology, high homology, and identical amino acids, respectively. (C, D) Cos-1 cells were transfected with expression vectors for TagRFP-EEA-1 and VN-H-Ras together with either wild-type (WT) or RAPEL-deleted (ΔRAPEL) PI3K RBD-VC vectors. Twenty-four hours after transfection, the cells were serum-starved for 4 hours and subjected to time-lapse confocal microscopy. At time 0, the cells were exposed to 100 ng/ml EGF. Representative images at 30 min after EGF stimulation are shown (C). Bar, 10 μm. The extent of colocalization of the Ras-PI3K complexes with EEA-1 was quantified as described in the Materials and Methods and plotted over time (D). Data are presented as the mean±s.e.m. (n≥20 from three independent experiments). *P*<0.0001 as calculated by MANOVA. (E, F) Cos-1 cells were transfected with expression vectors for HA-tagged wild-type (WT) or RAPEL-deleted (ΔRAPEL) PI3K p110γ. After 24 hours, the cells were subjected to immunofluorescence with the use of anti-EEA-1 and anti-HA antibodies. Representative images are shown (E). Left panels are higher magnification images of the inset indicated in the most right entire-cell images. Bar, 10 μm. The extent of colocalization of PI3K p110γ with EEA-1 was quantified (F). Data are presented as the mean±s.e.m. (*n*≥40 from three independent experiments). *P<0.0001 versus WT PI3K p110γ expressing cells as calculated by Welch’s *t*-tests. (G, H) 293T cells were transfected with a control vector (Ctrl) or an expression vector for either WT or ΔRAPEL PI3K RBD tagged with glutathione S-transferase (GST) together with a vector for Venus-H-Ras G12V (the constitutively active form). After 24 hours, the cells were subjected to pull-down assay, followed by immunoblotting with antibodies indicated at the right. An aliquot of the cell lysate was also analyzed as a loading control. Representative immunoblots are shown (G). Band intensities were quantitated and plotted (H). Data are means±s.e.m from three independent experiments. *P*=0.400 between WT and ΔRAPEL as calculated by Student’s *t*-test.

We have previously reported that the Ras-PI3K complex, but not complexes between Ras and other effectors, specifically localizes to endosomes (Tsutsumi *et al.*, 2009), which contributes to the regulation of clathrin-independent endocytosis and influenza virus infection (Fujioka *et al.*, 2011). To understand the molecular mechanism by which the Ras-PI3K complex is translocated to endosomes, RBD amino acid sequences were compared. The amino acid sequences of three major Ras effector families, including Raf (c-Raf, B-Raf, and A-Raf), PI3Ks (PI3K p110α, p110β, p110γ, and p110δ), and RalGEFs (RalGDS, Rlf, Rgl1, Rgl2, and Rgl3), were obtained from the National Center for Biotechnology Information (NCBI) Protein Database. The RBDs were identified by the NCBI Conserved Domain Database, and multiple sequence alignment was performed with the use of ClustalW (Fig. 1A and Fig. S1). A phylogenetic tree revealed that each family member forms distinct sister groups (same node), as expected. Interestingly, the PI3K group is the outgroup to Raf and RalGEF groups (Fig. S1), indicating that the RBD of PI3K is evolutionarily distinct from those of Raf and RalGEF. When the RBD sequences of PI3K p110α, p110β, and p110γ were directly compared with those of c-Raf and RalGDS, the PI3K RBDs were found to possess a unique N-terminal sequence of approximately 20 amino acids (Fig. 1A). Furthermore, 28-amino acid regions in the N-terminus of PI3K RBDs displayed high homology, leading us to examine whether this region plays a role in endosomal localization of the Ras-PI3K complex (Fig. 1B).

We thus prepared a PI3K p110γ RBD (simply termed PI3K RBD hereafter) that lacks these N-terminal 28 amino acids, and localization of the Ras-PI3K RBD complex was analyzed with the use of a bimolecular fluorescence complementation (BiFC) assay (Fig. 1C, D). In a quiescent state, Ras complexes with both wild-type and mutant PI3K RBD localized to the plasma membrane. Upon epidermal growth factor (EGF) stimulation, the Ras complex with wild-type RBD translocated to endosomes (Fig. 1C and Movie S1), as reported previously (Tsutsumi *et al.*, 2009). Quantitative analysis revealed that EGF promoted colocalization of the complex with early endosomes (visualized by early endosomal antigen-1 [EEA-1]) approximately 2-fold higher (at 30 min after EGF stimulation) than that in the unstimulated state (Fig. 1D). In contrast, the Ras complex with the mutant PI3K RBD was not retained in endosomes but rather accumulated in the perinuclear region (Fig. 1C, D, and Movie S2). The EGF-dependent recruitment of full-length PI3K p110γ (PI3Kγ) to endosomes was also inhibited by deletion of this region (Fig. 1E, F). Given that the mutant RBD could bind Ras to a similar extent as wild-type RBD (Fig. 1G, H), the N-terminal 28-amino acid region of RBD might possess a unique function in regulating localization of the Ras-PI3K complex without affecting its ability to bind Ras. The peptide (derived from PI3Kγ) was therefore named the Ras-PI3K endosomal localization (RAPEL) sequence.

### Inhibition of endocytosis and influenza virus infection by RAPEL expression

**Fig. 2:**
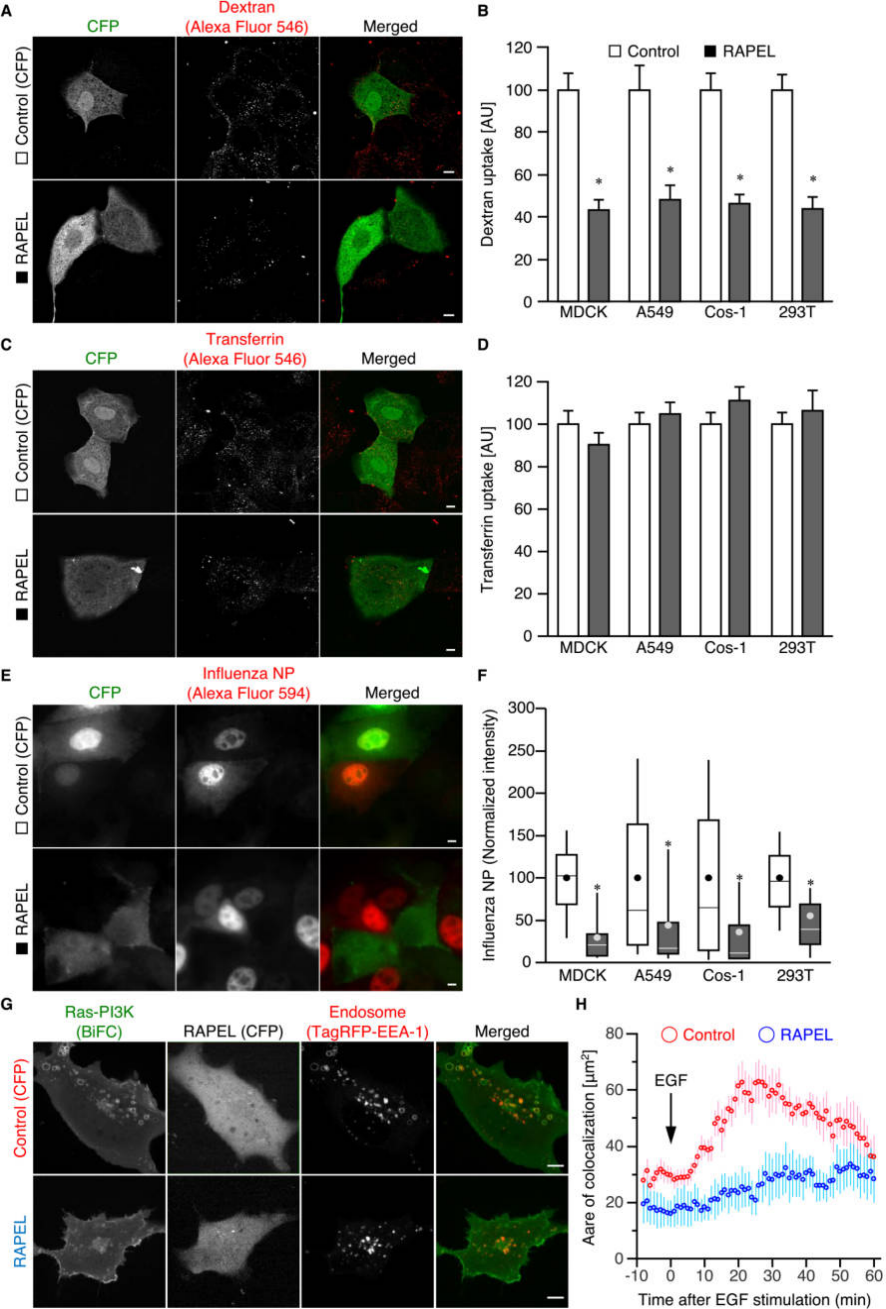
RAPEL expression inhibited endocytosis and IAV infection. (A–D) MDCK, A549, Cos-1, and 293T cells were transfected with expression vectors for CFP (Control) or CFP-RAPEL. Twenty-four hours after transfection, the cells were incubated with either Alexa Fluor 546-labeled dextran (10 kDa) for 10 min (A, B) or transferrin for 30 min (C, D) at 37°C and then washed with acidic buffer and PBS. Representative images of MDCK cells are shown (A, C). Bar, 10 μm. Total fluorescence intensity within cells was quantitated and plotted (B, D). Data are presented as the mean±s.e.m. (*n*≥30 from three independent experiments). *P<0.0001 versus control as calculated by Student’s *t*-tests. (E, F) MDCK, A549, Cos-1, and 293T cells were transfected with expression vectors for CFP or CFP-RAPEL. Twenty-four hours after transfection, the cells were infected with PR8 at an MOI of 1 PFU per cell for 4 hours and then subjected to an immunofluorescence-based virus infection assay. Representative images of MDCK cells are shown (E). Bar, 10 μm. Total fluorescence intensities within cells were quantitated for at least 55 cells in each of three independent experiments and plotted in box-and-whisker plots (F). *P<0.0001 versus control as calculated by Student’s *t*-tests. (G, H) Cos-1 cells were transfected with expression vectors for CFP or CFP-RAPEL together with those for TagRFP-EEA-1, VN-H-Ras, and wild-type PI3K RBD-VC. Twenty-four hours after transfection, the cells were serum-starved for 4 hours and subjected to time-lapse confocal microscopy. At time 0, the cells were exposed to 100 ng/ml EGF. Representative images at 30 min after EGF stimulation are shown (G). Bar, 10 μm. The extents of colocalization of the Ras-PI3K complexes with EEA-1 were quantified as described in the Materials and Methods and plotted over time (H). Data are presented as the mean±s.e.m. (*n*≥15 from three independent experiments). *P*=0.0004 as calculated by MANOVA.

Overexpression of a protein fragment that contains a regulatory domain often exerts a negative effect on downstream signaling by sequestering upstream factors, termed a dominant-negative effect. Thus, we next investigated whether the overexpression of RAPEL affects endocytosis. To evaluate the effects of RAPEL expression on clathrin-independent endocytosis at the single-cell level, cells were transfected with an expression vector harboring CFP-tagged RAPEL and challenged with fluorescently labeled 10-kDa dextran (Fig. 2A, B) and 70-kDa dextran (Fig. S2A, B) that were reported to enter cells via different clathrin-independent pathways (Li *et al.*, 2015). As expected, RAPEL expression significantly inhibited the uptake of both dextrans in MDCK cells (Fig. 2A, B, and Fig. S2A, B). This inhibition was also observed in A549, Cos-1, and 293T cells (Fig. 2A, B), indicating that the inhibitory effect of RAPEL on endocytosis is functional among several cell contexts. In contrast, RAPEL expression did not affect transferrin uptake (Fig. 2C, D), demonstrating that RAPEL plays a role in the regulation of clathrin-independent endocytosis, including macropinocytosis and micropinocytosis, but not in clathrin-mediated endocytosis. This notion is consistent with our previous report that Ras-PI3K signaling is preferentially involved in the regulation of clathrin-independent endocytosis (Fujioka *et al.*, 2011).

We have previously reported that influenza A virus (IAV) enters host cells via both clathrin-mediated and clathrin-independent endocytosis (Fujioka *et al.*, 2013), and Ras-PI3K signaling plays a role in virus internalization and infection (Fujioka *et al.*, 2011). Therefore, we investigated whether RAPEL overexpression inhibited IAV infection. An MDCK plaque assay revealed that RAPEL overexpression suppressed the plaque-forming activity of the IAV strain A/Puerto Rico/8/34 (H1N1; PR8) in 293T cells (Fig. S2C, D). An essentially similar inhibitory effect of RAPEL on replication of another IAV strain A/Aichi/2/68 (H3N2; Aichi) was observed (Fig. S2E). Next, to further examine the effect of RAPEL expression on the infection in other cell lines, we utilized immunofluorescence-based infection assay. This assay allows for visualization of single-round replication of IAV at a single cell level (Fujioka *et al.*, 2018), and the percentage of nucleoprotein (NP)-positive cells is well correlated with the titer of infected viruses (Fig. S2F, compare with Fig. S2C) (Fujioka *et al.*, 2018). It was thereby revealed that IAV infection was inhibited by RAPEL expression in A549, Cos-1, and 293T cells, in addition to MDCK cells (Fig. 2E, F). Given that recruitment of the Ras-PI3K complex to endosomes plays a role in IAV internalization (Fujioka *et al.*, 2011), RAPEL is thought to hamper influenza virus infection as an inhibitor of the virus internalization step. Indeed, RAPEL overexpression also inhibited virus internalization (Fig. S2G). Moreover, endosomal localization of the Ras complex with wild-type PI3K RBD was inhibited in RAPEL-expressing cells, as visualized by BiFC (Fig. 2G, H). Notably, RAPEL expression did not affect EGF-induced Ras activation (Fig. S3A), mitogen-activated protein kinase/extracellular signal-regulated kinase (MAPK/ERK) phosphorylation, or Akt phosphorylation (Fig. S3B–D). Therefore, RAPEL might specifically impact Ras-PI3K localization and inhibit endocytosis without affecting Ras or its downstream signaling emitted at the plasma membrane.

### Determination of the minimal essential and critical residues in RAPEL

**Fig. 3:**
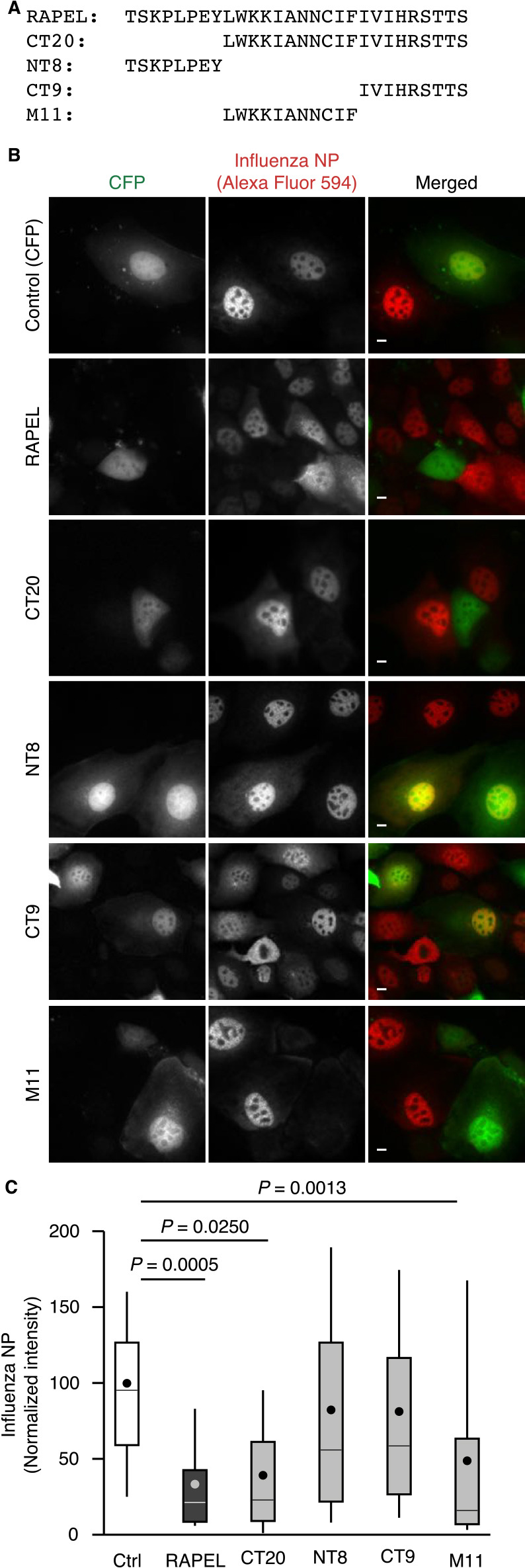
Determination of the minimal peptide required for RAPEL function. (A) Amino acid sequences of full-length RAPEL and truncated RAPEL mutants used in this study. (B, C) MDCK cells were transfected with expression vectors for the proteins indicated on the left (B) or at the bottom (C). After 24 hours, the cells were infected with PR8 at an MOI of 1 PFU per cell for 4 hours and then subjected to an immunofluorescence-based virus infection assay. Representative images are shown (B). Bar, 10 μm. Total fluorescence intensity of NP within cells was quantitated for at least 80 cells in each of three independent experiments and plotted in box-and-whisker plots (C). *P* values were calculated by one-way ANOVA with post-hoc Tukey HSD test and are shown.

We next aimed to minimize the peptide length of RAPEL aiming at its facile synthesis and thus prepared a variety of RAPEL truncation mutants (Fig. 3A). Expression of peptides with an 8-amino acid deletion at the N-terminus (CT20) inhibited virus infection to a similar extent as full-length RAPEL (Fig. 3B, C), whereas expression of 8 N-terminal amino acids and 9 C-terminal amino acids (NT8 and CT9) failed to do so, indicating that the middle 11 amino acids (M11) are required for RAPEL function. In fact, M11 expression inhibited virus infection to an extent comparable to that of full-length RAPEL (Fig. 3B, C).

**Fig. 4:**
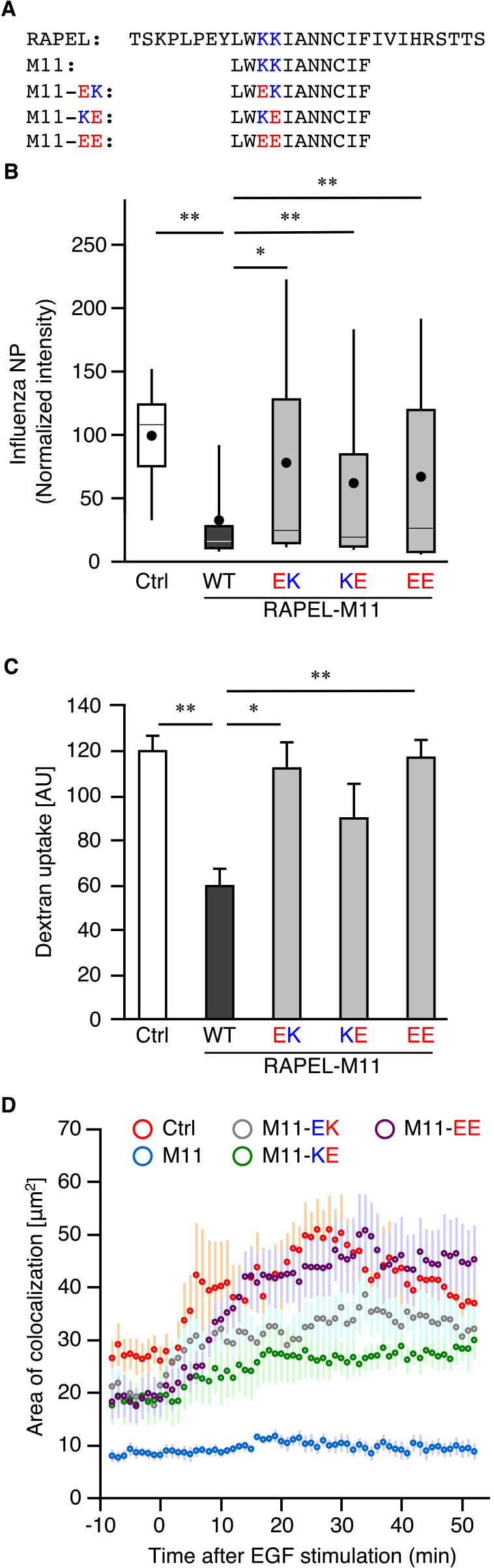
Lysine residues in RAPEL were crucial for its function. (A) Amino acid sequences of RAPEL-M11 and RAPEL-M11 mutants in which one or two lysine residues were replaced with glutamate. (B–D) Cos-1 cells were transfected with expression vectors for CFP (Ctrl), CFP-RAPEL-M11, or CFP-RAPEL-M11 mutant as indicated. In (D), the cells were further transfected with expression vectors for VN-H-Ras and wild-type PI3K RBD-VC. After 24 hours, the cells were exposed to PR8 at an MOI of 1 PFU per cell for 4 hours (B), to Alexa Fluor 546-conjugated dextran for 10 min (C), or to 100 ng/ml EGF for 50 min (D). The cells were then subjected to infection (B), endocytosis (C), and BiFC (D) assays. The fluorescence intensities of NP (B) and dextran (C) were quantitated and plotted. *P<0.002 and **P<0.0001 as calculated by one-way ANOVA with post-hoc Tukey HSD test. In (D), intracellular granules formed by the BiFC signal with EEA-1 were quantified as described in the Materials and Methods and plotted over time. Data are presented as the mean±s.e.m. (*n*≥10 from three independent experiments). *P*<0.0001 between control and M11; *P*=0.842 between control and EK; *P*=0.0959 between control and KE; *P*=0.4055 between control and EE; *P*=0.0006 between M11 and KE, as calculated by MANOVA and Bonferroni correction. See Fig. S4 for imaging data used to obtain the quantitative data shown here.

We further aimed to identify amino acids that are critical for the inhibitory effect of RAPEL on endocytosis and virus infection. The middle region of RAPEL (M11) consists of 7 nonpolar amino acids, two basic amino acids (lysine), and two polar amino acids (asparagine) (Fig. 4A). In addition, one of the two lysine residues is conserved among p110α, p110β, and p110γ (see Fig. 1B). Therefore, we decided to substitute this lysine with the acidic amino acid glutamate (Fig. 4A). All mutant RAPEL peptides (M11-EK, M11-KE, and M11-EE) in which either or both lysine residues were substituted by glutamate partially lost the ability to inhibit influenza virus infection (Fig. 4B and Fig. S4A). These RAPEL M11 mutants also failed to inhibit clathrin-independent endocytosis (Fig. 4C and Fig. S4B) and endosomal localization of the Ras-PI3K complex (Fig. 4D and Fig. S4C). These results indicated that lysine residues within RAPEL are critical for endosomal localization of the Ras-PI3K complex and the subsequent regulation of clathrin-independent endocytosis. Therefore, substituting the lysine residues with glutamate resulted in a decreased ability to inhibit endocytosis and influenza virus infection.

To further examine whether RAPEL sequence and the lysine residues contained therein are required for IAV internalization, we performed rescue experiments, in which wild-type PI3K or its mutants were introduced into embryonic fibroblasts from mice deficient in PIK3CG, the gene encoding the catalytic subunit of PI3Kγ (p110γ) (Fujioka *et al.*, 2011; Sasaki *et al.*, 2000). Constructed PI3K mutants include PI3K in which either or both lysine residues were replaced with asparagine (PI3K-EK, PI3K-KE, and PI3K-EE), in addition to PI3K lacking RAPEL sequence (PI3K-ΔRAPEL). Whereas wild-type PI3K restored IAV internalization in PI3K-knockout mouse embryonic fibroblasts, which is consistent with our previous report (Fujioka *et al.*, 2011), the mutants failed to do so (Fig. S5). These results indicated that RAPEL sequence, particularly the lysine residues in RAPEL, is involved in the regulation of IAV infection at the entry step.

### RAPEL as a peptide therapeutic

**Fig. 5:**
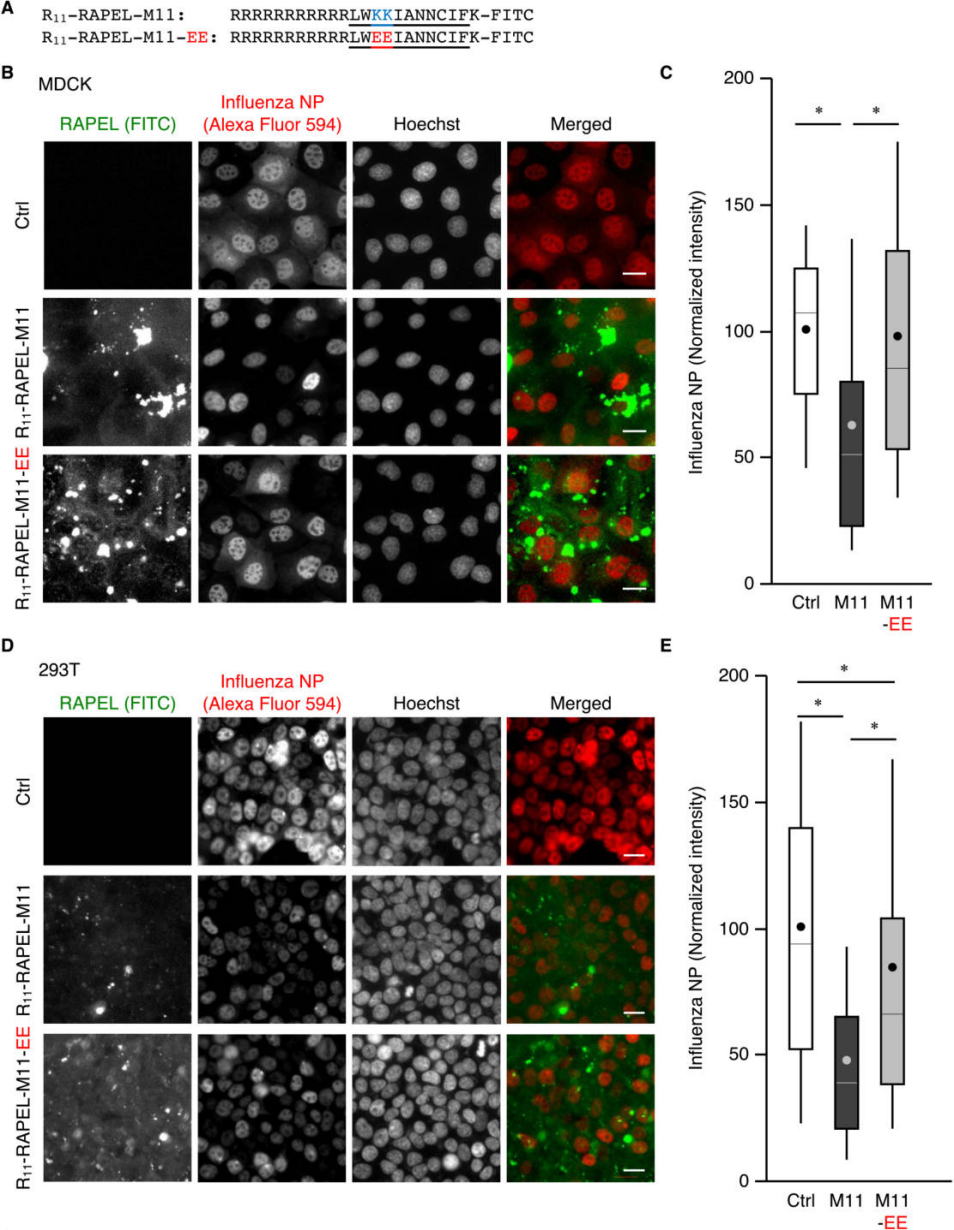
Introduction of RAPEL as a synthetic peptide inhibited IAV infection. (A) Amino acid sequences of the synthetic peptides R_11_-RAPEL-M11 and R_11_-RAPEL-M11-EE. FITC, fluorescein isothiocyanate. (B–E) MDCK (B, C) and 293T (D, E) cells were grown to 80% confluency, treated with 100 μM R_11_-RAPEL-M11 or R_11_-RAPEL-M11-EE or left untreated for 1 hour, and after washing, further incubated for 24 hours. The cells were then infected with PR8 at an MOI of 1 PFU per cell for 4 hours and subjected to an immunofluorescence-based infection assay. Representative images are shown (B, D). Bar, 10 μm. The fluorescence intensities of NP were quantitated for at least 180 cells for MDCK and 1,400 cells for 293T in each of three independent experiments and plotted in box-and-whisker plots (C, E). *P<0.0001 as calculated by one-way ANOVA with post-hoc Tukey HSD test.

We next examined whether RAPEL can be applied as a peptide therapeutic using cell-penetrating peptides (CPPs). The synthetic peptides consisted of 11 arginine residues (R_11_) presented as CPP, RAPEL-M11 (either wild type or EE), and an extra lysine residue for the covalent conjugation of a fluorescent dye (fluorescein-5-isothiocyanate, FITC) from the N-terminus (Fig. 5A). Treatment of cells with these peptides resulted in cytoplasmic delivery and accumulation to the perinuclear region to some extent, as visualized by fluorescence microscopy (Fig. 5B, D, and Fig. S6A). When MDCK cells were treated with R_11_-RAPEL-M11, IAV infection was modestly inhibited, as detected by NP staining (Fig. 5B, C). Similarly, R_11_-RAPEL-M11 also inhibited IAV infection in 293T cells significantly (Fig. 5D, E). In contrast, R_11_-RAPEL-M11-EE did not significantly impact IAV infection in MDCK cells (Fig. 5B, C). This EE mutant inhibited the infection to some extent in 293T cells; however, this inhibitory effect was significantly less effective than that of wild-type R_11_-RAPEL-M11 (Fig. 5D, E). Essentially similar results were obtained when we evaluated the effect of RAPEL peptides on the titer of infectious virus particles by plaque assay (Fig. S6B and C).

We further investigated the effect of RAPEL peptide on IAV infection in an ex vivo model with the use of the human respiratory epithelial cell line BEAS-2B. When cultured in Matrigel, these cells form a monolayer that mimics bronchial epithelium (Fujioka *et al.*, 2018; Shen *et al.*, 2015). Such epithelial monolayers were pretreated with RAPEL peptides and exposed to PR8. Whereas treatment with R_11_-RAPEL-M11 inhibited IAV infection, that with R_11_-RAPEL-M11-EE had no significant inhibitory effect on the infection (Fig. S7). Together, these results demonstrate the significance of RAPEL-M11 with CPPs for peptide therapy applications against IAV.

### Participation of PI3P in the regulation of endosomal localization of the Ras-PI3K complex

**Fig. 6:**
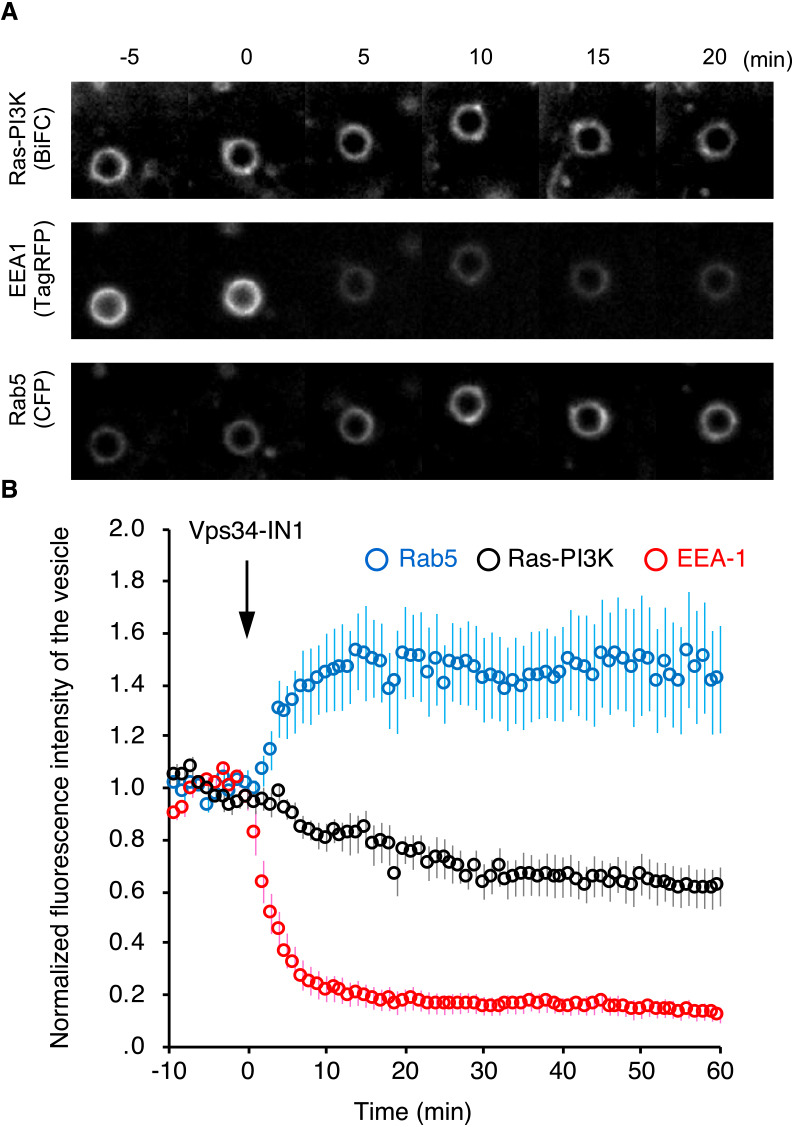
PI3P is required to endosomal localization of the Ras-PI3K complex. Cos-1 cells were transfected with expression vectors for TagRFP-EEA-1, CFP-Rab5, and VN-H-Ras G12V. Twenty-four hours after transfection, the cells were subjected to time-lapse confocal microscopy. At time 0, the cells were exposed to Vps34-IN1. Representative images are shown (A). Bar, 2 μm. The fluorescence intensities of NP within cell were quantified and plotted over time (B). Data are presented as the mean±s.e.m. (*n*=12 from three independent experiments). *P*<0.0001 compared with before treatment, as calculated by repeated-measures ANOVA.

Finally, we endeavored to explore the molecular mechanism by which the Ras-PI3K complex is translocated to the endosome via RAPEL sequence. To do this, the effect of inhibitor of the class III PI3K Vps34 (Vps34-IN1) on Ras-PI3K translocation. Vps34 is an enzyme that produces phosphatidylinositol 3-phosphate (PI3P, the most abundant phosphoinositide on the cytoplasmic leaflets of the early endosomal membrane) (Lemmon, 2008). The treatment with Vps34-IN1 partially, but significantly reduced the amount of the Ras-PI3K complex in the endosome, as visualized by BiFC (Fig. 6). Under this condition, the fluorescence signal in endosomes of RFP-tagged EEA-1, which is recruited to the endosomes through interaction with PI3P (Law *et al.*, 2017), was dramatically dispersed by the treatment. In contrast, CFP-tagged Rab5, which is deactivated and dispersed by PI3P production, accumulated in the endosome, confirming PI3P depletion on the endosomal membrane. Taken together, PI3P might be involved, albeit in part, in RAPEL-dependent localization of the Ras-PI3K complex in the endosome.

## Discussion

In this study, we identified RAPEL as the region of PI3K that is important for endosomal localization of the Ras-PI3K complex. Deletion of this region resulted in an inability of the complex to be retained in the endosome and was instead fast trafficked to the perinuclear region (recycling endosomes). Overexpression of RAPEL inhibited clathrin-independent endocytosis as well as IAV internalization and infection. In total, 11 amino acids were deemed essential for primary RAPEL function, among which the lysine residues appeared to play a role in the dominant-negative effect of RAPEL. IAV infection was inhibited by not only RAPEL expression as a genetically encoded peptide but also by its application in peptide form, demonstrating the significance of RAPEL as a therapeutic agent.

Endocytosis, a fundamental cellular mechanism, is involved in a wide range of pathophysiological phenomena, including substance uptake, receptor downregulation, epithelial polarity, barrier function maintenance, microbial infection, and apoptotic cell phagocytosis (Miller and Krijnse-Locker, 2008). Lipid content, particularly that of phosphatidylinositols, at endosomal membranes is specific for the compartment. The relative abundances of phosphatidylinositols are altered during endosomal maturation, which thereby recruits different proteins to different endosomal components. The most abundant phosphatidylinositol in endosomes is PI3P, which promotes the endosomal localization of EEA-1, a marker for early endosomes. This phosphoinositide also participated in the recruitment of Ras-PI3K complex (Fig. 6); however, it remains controversial whether the enzymatic activity of type I PI3K is indeed required for this phenomenon. The requirement for type I PI3K enzymatic activity in endosomes is supported by reports that switching from phosphatidylinositol 4,5-bisphosphate (PIP_2_) to phosphatidylinositol 3,4,5-trisphosphate (PIP_3_) is crucial for the maturation of macropinocytosis and that the subsequent loss of PIP_3_ promotes the acquisition of PI3P (Porat-Shliom *et al.*, 2008; Zoncu *et al.*, 2009). On the other hand, the possibility of PI3K functioning as something other than a lipid kinase, such as a scaffold protein, may also play a role therein. Future work will be needed to clarify this possibility.

Although we demonstrated the requirement for RAPEL in recruiting (retaining) the Ras-PI3K complex to the endosome, the molecular mechanism by which this complex is recruited via RAPEL remains unknown. A possible mechanism is that molecule(s) that binds to RAPEL in the endosome tethers the Ras-PI3K complex to the endosome (Fig. S8). Overexpression of RAPEL might sequester this molecule, thereby inhibiting endocytosis and IAV infection. Such a molecule is not necessarily localized to endosomes in quiescent conditions but is rather transiently translocated in a manner dependent on stimulation. Screening the molecules that bind to RAPEL in the endosomes, would be a useful approach for answering these questions in the future.

## Acknowledgments

We thank A. Miyawaki for providing Venus cDNA, Len Stephen for providing p110γ cDNA, and A. Kikuchi for technical assistance. This work was supported in part by Grants-in-Aid from the Ministry of Education, Culture, Sports, Science and Technology of Japan (#26115701 and #15H01248) and the Japan Society for the Promotion of Science (#26293041 and #16H06227) as well as by grants from the Mochida Memorial Foundation for Medical and Pharmaceutical Research, the Waksman Foundation of Japan, the Sumitomo Electric Group Corporate Social Responsibility Foundation and the SENSHIN Medical Research Foundation.

## Conflict of interest

The authors declare that there is no conflict of interest.

## Supplementary Material

Fig. S1 Comparison of amino acid sequences of RBDs of Ras effectors. Amino acid sequences of the RBDs of Ras effecter molecules were analyzed by ClustalW, and a rooted phylogenetic tree (UPGMA) was drawn. The accession number for each molecule is provided to the right of each molecular name.

Fig. S2 RAPEL expression inhibited endocytosis, IAV infection, and internalization. (A) MDCK cells were transfected with expression vectors for CFP (Control) or CFP-RAPEL. After 24 hours, the cells were incubated with rhodamine B-conjugated dextran (70-kD) for 10 min at 37°C and then washed with acidic buffer and PBS. Representative images are shown (A). Bar, 10 μm. Total fluorescence intensity within cells was quantitated and plotted (B). Data are presented as the mean±s.e.m. (*n*≥30 from three independent experiments). **P*<0.0001 versus control as calculated by Student’s *t*-tests. (C) 293T cells expressing CFP-RAPEL or CFP (Control) were infected with PR8 at an MOI of 1 PFU per cell for 1 hour. The titer of infectious virus particles released into culture medium was determined with an MDCK plaque assay at 12, 24, 36, and 48 hours post infection (hpi). (D, E) Replication of PR8 (D) or Aichi (E) in 293T cells expressing CFP-RAPEL or CFP (Control) was determined with an MDCK plaque assay at 48 hpi. Data are presented as the mean±s.e.m. from three independent experiments. **P*=0.014 (D) and *P*=0.012 (E) versus control as calculated by Student’s *t*-tests. (F) MDCK cells expressing CFP-RAPEL or CFP (Control) were infected with PR8 at an MOI of 1 PFU per cell for indicated time periods and then subjected to an immunofluorescence-based virus infection assay. Total fluorescence intensities within cells were quantitated and plotted. Data are presented as the mean±s.e.m. (*n*≥15 from three independent experiments). **P*<0.006 versus control as calculated by Welch’s *t*-test with Bonferroni correction. (G) MDCK cells expressing CFP-RAPEL or CFP (Control) were infected with PR8 at an MOI of 10 PFU per cell for 1 hour. The cells were then subjected to immunofluorescence-based internalization assay as described in the Materials and Methods. Rab7- and NP-positive regions were extracted and their colocalization was determined. Data are means±s.e.m. (n≥20). **P*=0.0035 versus control cells, as calculated by Student’s *t*-test.

Fig. S3 RAPEL is dispensable for canonical Ras signaling. (A) Cos-1 cells were transfected with expression vectors for mRFP or mRFP-RAPEL with Raichu-Ras for 24 hours and then subjected to time-lapse microscopy. The cells were stimulated with 100 ng/ml EGF at time 0. Normalized emission ratio (FRET/CFP), which represent Ras activity were calculated and plotted. Data are means±s.e.m. (*n*≥15 from three independent experiments). *P*=0.6074 as calculated by MANOVA. (B–D) Cos-1 cells were transfected with expression vectors for CFP (control) or CFP-RAPEL (RAPEL) for 24 hours. The cells were then stimulated with 100 ng/ml EGF for 30 min, lysed in lysis buffer, and subjected to immunoblotting with the use of the indicated antibodies. Representative immunoblots are shown (B). Band intensities were quantitated and plotted (C–D). Data are means±s.e.m. from three independent experiments. *P* values were calculated by one-way ANOVA with post-hoc Tukey HSD test and are shown.

Fig. S4 Lysine residues in RAPEL are crucial for its function. Representative imaging data used for quantitative analyses in Figs. 4B (A), 4C (B), and 4D (C) are shown. Bar, 10 μm.

Fig. S5 Lysine residues in RAPEL are crucial for virus internalization. (A) Embryonic fibroblasts from wild-type mice or derivatives of those from mice deficient in *PIK3CG* were prepared as described in the Materials and Methods. The cells were infected with PR8 at an MOI of 10 PFU per cell for 1 hour and then subjected to an immunofluorescence-based virus internalization assay. Rab7- and NP-positive regions were extracted and their colocalization was determined and plotted. Data are means±s.e.m. (*n*≥50 from three independent experiments). **P*<0.0001 as calculated by one-way ANOVA with post-hoc Tukey HSD test. (B) Total cell lysates obtained from each cell line were subjected to SDS-PAGE, followed by immunoblotting with an anti-HA antibody. Actin was used as a loading control. Representative immunoblots from three independent experiments are shown.

Fig. S6 Introduction of RAPEL peptide inhibited IAV infection. (A) The images of peptide in Figs. 5B and D were displayed in “FIRE” pseudo color mode. (B–C) MDCK (B) and 293T (C) cells were grown to 80% confluency, treated with 100 μM R_11_-RAPEL-M11 or R_11_-RAPEL-M11-EE or left untreated for 1 hour, and after washing, further incubated for 24 hours. The cells were then infected with PR8 at an MOI of 1 PFU per cell for 48 hours and subjected to an MDCK plaque assay. **P*<0.01 as calculated by one-way ANOVA with post-hoc Tukey HSD test.

Fig. S7 Introduction of RAPEL peptide inhibited IAV infection ex vivo. (A, B) Human bronchial epithelial cells (BEAS-2B) were cultured in Matrigel. After formation of an epithelial monolayer, the cells were incubated with 100 μM R_11_-RAPEL-M11 or R_11_-RAPEL-M11-EE or left untreated for 1 hour, and after washing, further incubated for 24 hours. The cells were then infected with PR8 at an MOI of 1 PFU per cell for 4 hours and subjected to immunofluorescence for NP. Nuclei were counterstained with Hoechst 33342. 3D images reconstructed from confocal images covering from the top to the bottom of the monolayers and are shown (A). Bar, 10 μm. Selected confocal images were subjected to the immunofluorescence infection assay to quantitate total fluorescence intensities of NP within each individual cell (B). Data are presented as the mean±s.e.m. (*n*>7000 from three independent experiments). **P*<0.0001 as calculated by one-way ANOVA with post-hoc Tukey HSD test.

Fig. S8 A Model for the inhibitory mechanism of influenza virus entry by RAPEL overexpression or RAPEL peptide treatment.

Movie S1 Representative video images of the complex between Ras and wild-type PI3K RBD in the presence of EGF (related to Fig. 1C, D). Cos-1 cells expressing TagRFP-EEA-1, VN-H-Ras WT, and wild-type PI3K RBD-VC exposed to EGF were monitored by an sDISK spinning disk confocal microscope.

Movie S2 Representative video images of the complex between Ras and PI3K RBD deficient in RAPEL in the presence of EGF (related to Fig. 1C, D). Cos-1 cells expressing TagRFP-EEA-1, VN-H-Ras WT, and ΔRAPEL PI3K RBD-VC exposed to EGF were monitored by an sDISK spinning disk confocal microscope.
