# Supplementary figures and images for: A Peptide Derived from Phosphoinositide 3-kinase Inhibits Endocytosis and Influenza Virus Infection
Source: Cell Struct Funct. 2019 Mar 21;44(1):61–74. doi: 10.1247/csf.19001 (PMC11926411; doi:10.1247/csf.19001)

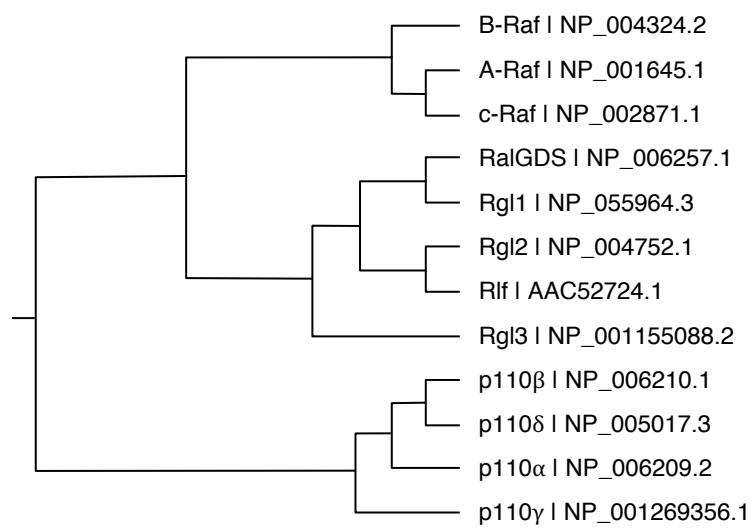

Fig. S1 Fujioka, Satoh et al.

Supplement: Supplementary file 1 — Fig. S1 Comparison of amino acid sequences of RBDs of Ras effectors. Amino acid sequences of the RBDs of Ras effecter molecules were analyzed by ClustalW, and a rooted phylogenetic tree (UPGMA) was drawn. The accession number for each molecule is provided to the right of each molecular name. [file csf_44_19001_1.pdf]

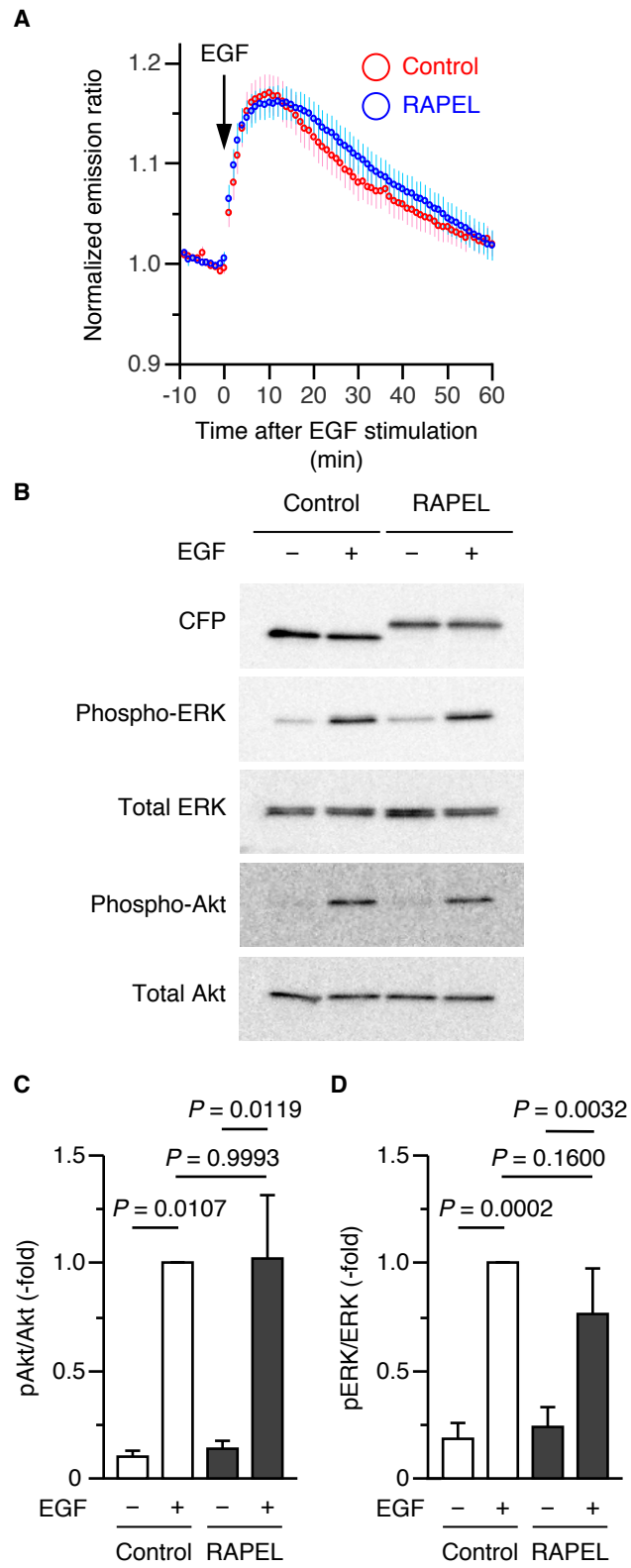

Fig. S3 Fujioka, Satoh et al.

Supplement: Supplementary file 3 — Fig. S3 RAPEL is dispensable for canonical Ras signaling. (A) Cos-1 cells were transfected with expression vectors for mRFP or mRFP-RAPEL with Raichu-Ras for 24 hours and then subjected to time-lapse microscopy. The cells were stimulated with 100 ng/ml EGF at time 0. Normalized emission ratio (FRET/CFP), which represent Ras activity were calculated and plotted. Data are means±s.e.m. (n≥15 from three independent experiments). P=0.6074 as calculated by MANOVA. (B–D) Cos-1 cells were transfected with expression vectors for CFP (control) or CFP-RAPEL (RAPEL) for 24 hours. The cells were then stimulated with 100 ng/ml EGF for 30 min, lysed in lysis buffer, and subjected to immunoblotting with the use of the indicated antibodies. Representative immunoblots are shown (B). Band intensities were quantitated and plotted (C–D). Data are means±s.e.m. from three independent experiments. P values were calculated by one-way ANOVA with post-hoc Tukey HSD test and are shown. [file csf_44_19001_3.pdf]

**A** Images for Fig. 4B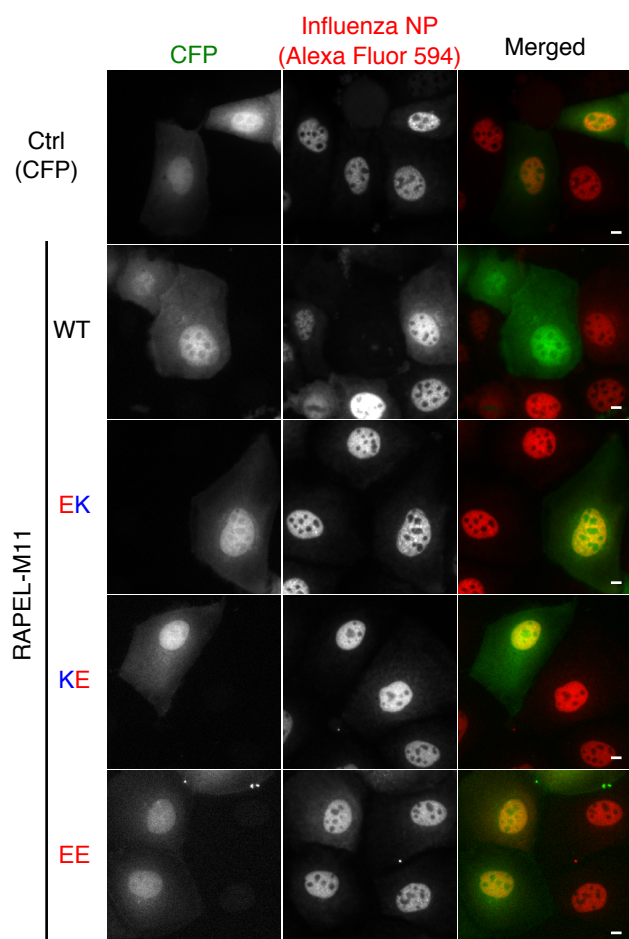**B** Images for Fig. 4C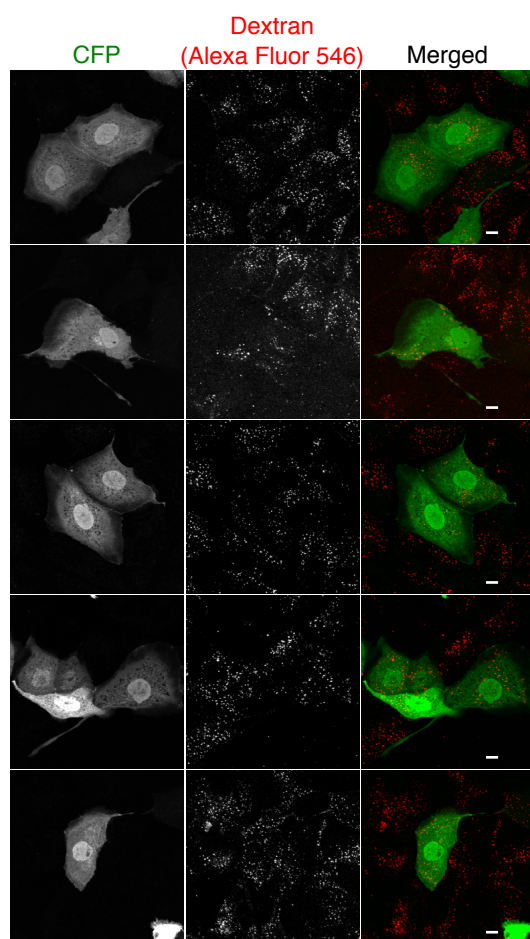**C** Images for Fig. 4D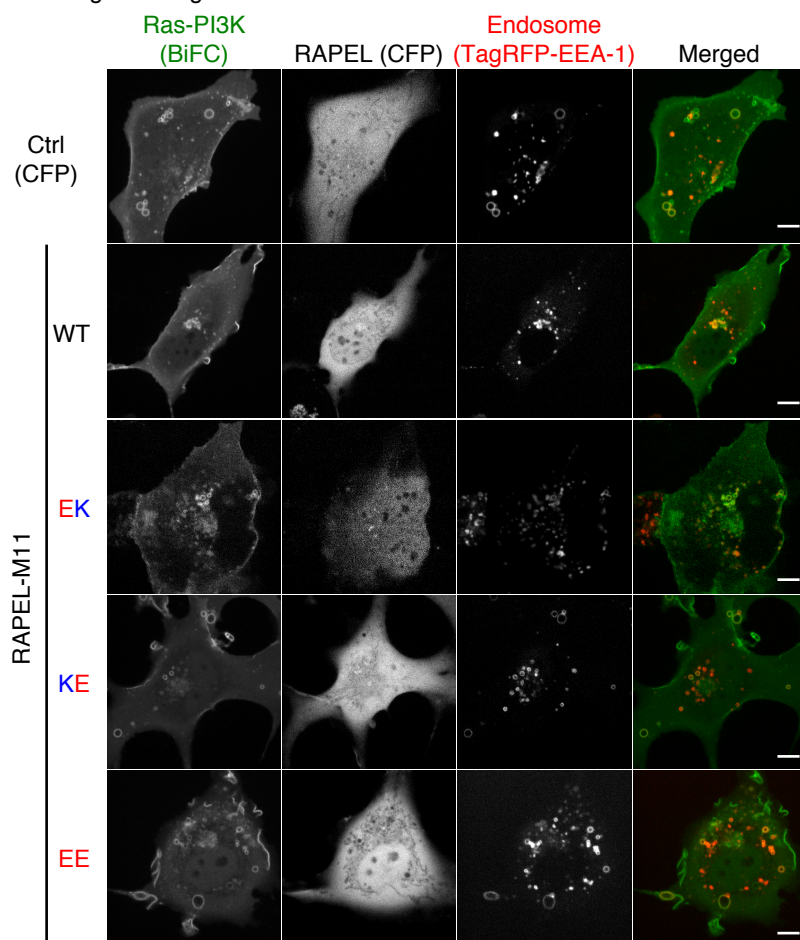

Supplement: Supplementary file 4 — Fig. S4 Lysine residues in RAPEL are crucial for its function. Representative imaging data used for quantitative analyses in Figs. 4B (A), 4C (B), and 4D (C) are shown. Bar, 10 μm. [file csf_44_19001_4.pdf]

**A**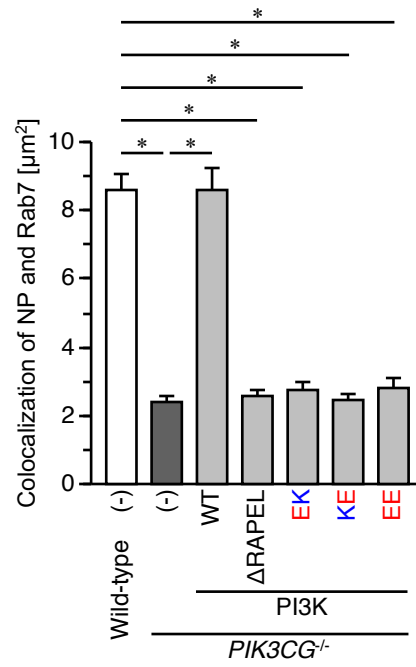**B**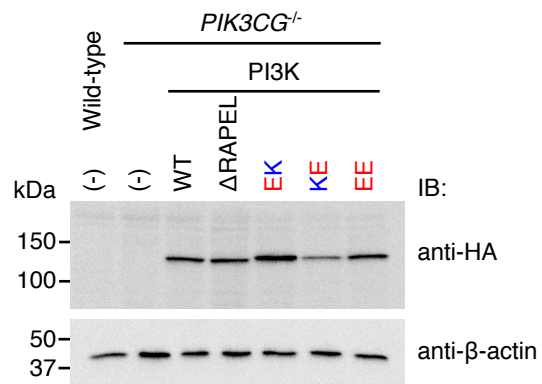

Supplement: Supplementary file 5 — Fig. S5 Lysine residues in RAPEL are crucial for virus internalization. (A) Embryonic fibroblasts from wild-type mice or derivatives of those from mice deficient in PIK3CG were prepared as described in the Materials and Methods. The cells were infected with PR8 at an MOI of 10 PFU per cell for 1 hour and then subjected to an immunofluorescence-based virus internalization assay. Rab7- and NP-positive regions were extracted and their colocalization was determined and plotted. Data are means±s.e.m. (n≥50 from three independent experiments). *P<0.0001 as calculated by one-way ANOVA with post-hoc Tukey HSD test. (B) Total cell lysates obtained from each cell line were subjected to SDS-PAGE, followed by immunoblotting with an anti-HA antibody. Actin was used as a loading control. Representative immunoblots from three independent experiments are shown. [file csf_44_19001_5.pdf]

**A**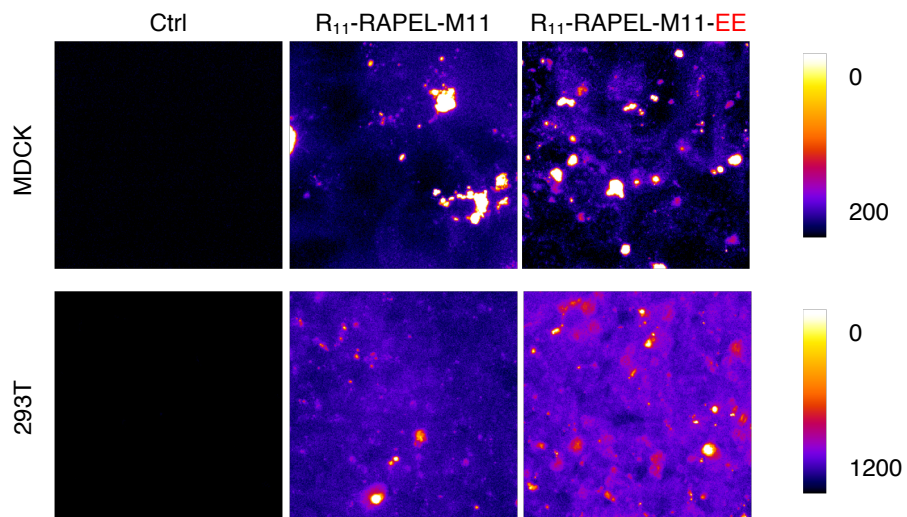**B**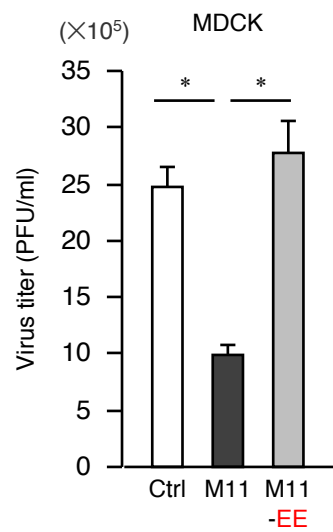**C**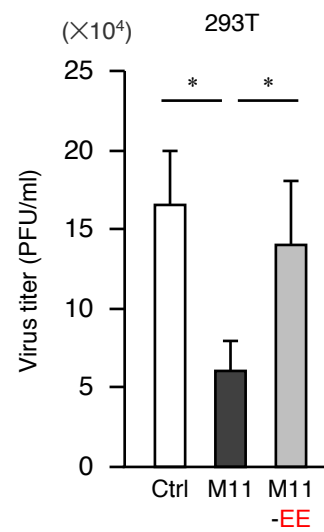

Supplement: Supplementary file 6 — Fig. S6 Introduction of RAPEL peptide inhibited IAV infection. (A) The images of peptide in Figs. 5B and D were displayed in “FIRE” pseudo color mode. (B–C) MDCK (B) and 293T (C) cells were grown to 80% confluency, treated with 100 μM R11-RAPEL-M11 or R11-RAPEL-M11-EE or left untreated for 1 hour, and after washing, further incubated for 24 hours. The cells were then infected with PR8 at an MOI of 1 PFU per cell for 48 hours and subjected to an MDCK plaque assay. *P<0.01 as calculated by one-way ANOVA with post-hoc Tukey HSD test. [file csf_44_19001_6.pdf]

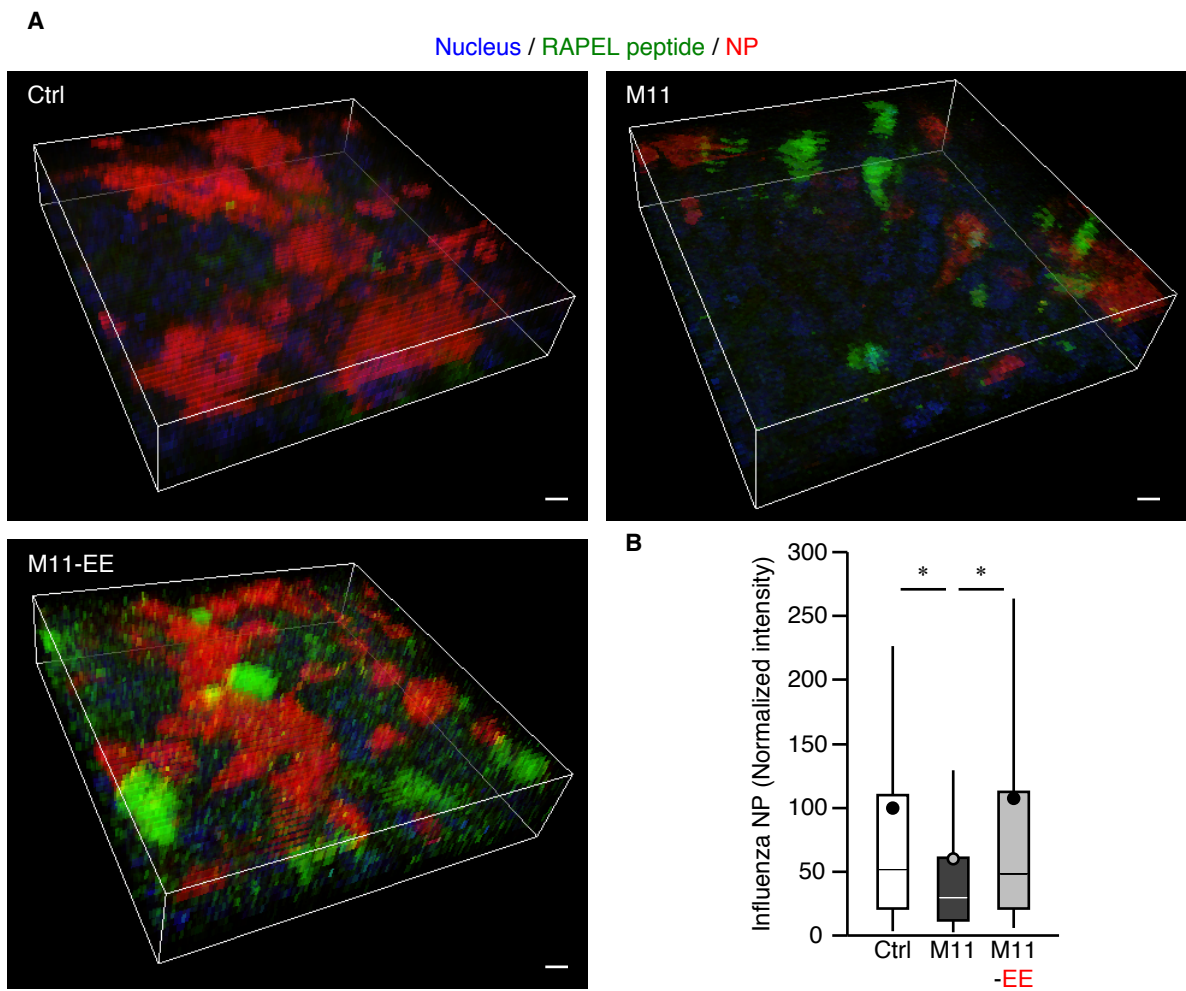

Fig. S7 Fujioka, Satoh et al.

Supplement: Supplementary file 7 — Fig. S7 Introduction of RAPEL peptide inhibited IAV infection ex vivo. (A, B) Human bronchial epithelial cells (BEAS-2B) were cultured in Matrigel. After formation of an epithelial monolayer, the cells were incubated with 100 μM R11-RAPEL-M11 or R11-RAPEL-M11-EE or left untreated for 1 hour, and after washing, further incubated for 24 hours. The cells were then infected with PR8 at an MOI of 1 PFU per cell for 4 hours and subjected to immunofluorescence for NP. Nuclei were counterstained with Hoechst 33342. 3D images reconstructed from confocal images covering from the top to the bottom of the monolayers and are shown (A). Bar, 10 μm. Selected confocal images were subjected to the immunofluorescence infection assay to quantitate total fluorescence intensities of NP within each individual cell (B). Data are presented as the mean±s.e.m. (n>7000 from three independent experiments). *P<0.0001 as calculated by one-way ANOVA with post-hoc Tukey HSD test. [file csf_44_19001_7.pdf]

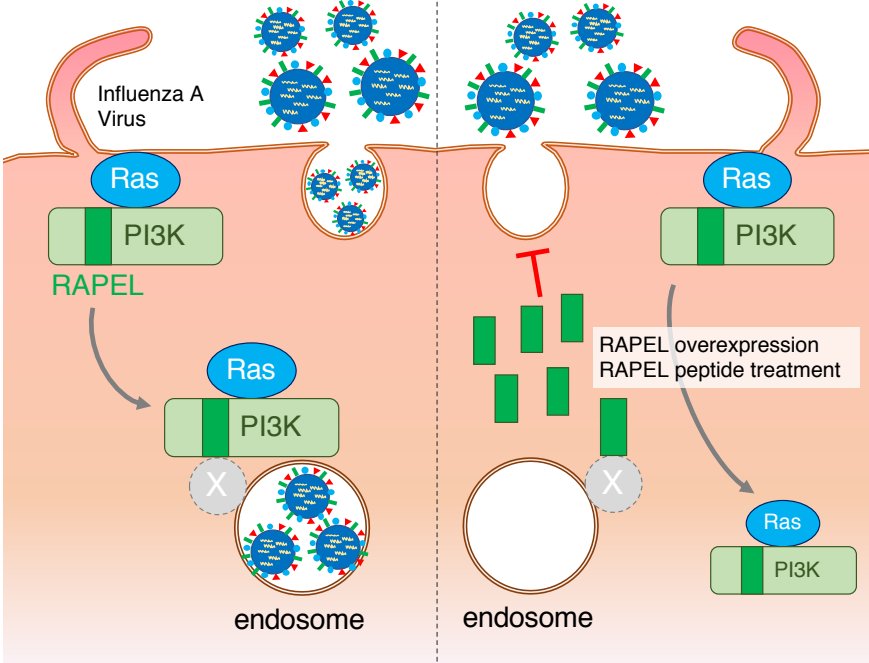

Fig. S8 Fujioka, Satoh et al.

Supplement: Supplementary file 8 — Fig. S8 A Model for the inhibitory mechanism of influenza virus entry by RAPEL overexpression or RAPEL peptide treatment. [file csf_44_19001_8.pdf]
