# Supplementary material for: A Peptide Derived from Phosphoinositide 3-kinase Inhibits Endocytosis and Influenza Virus Infection
Source: Cell Struct Funct. 2019 Mar 21;44(1):61–74. doi: 10.1247/csf.19001 (PMC11926411; doi:10.1247/csf.19001)
Supplement: Supplementary file 2 — Fig. S2 RAPEL expression inhibited endocytosis, IAV infection, and internalization. (A) MDCK cells were transfected with expression vectors for CFP (Control) or CFP-RAPEL. After 24 hours, the cells were incubated with rhodamine B-conjugated dextran (70-kD) for 10 min at 37°C and then washed with acidic buffer and PBS. Representative images are shown (A). Bar, 10 μm. Total fluorescence intensity within cells was quantitated and plotted (B). Data are presented as the mean±s.e.m. (n≥30 from three independent experiments). *P<0.0001 versus control as calculated by Student’s t-tests. (C) 293T cells expressing CFP-RAPEL or CFP (Control) were infected with PR8 at an MOI of 1 PFU per cell for 1 hour. The titer of infectious virus particles released into culture medium was determined with an MDCK plaque assay at 12, 24, 36, and 48 hours post infection (hpi). (D, E) Replication of PR8 (D) or Aichi (E) in 293T cells expressing CFP-RAPEL or CFP (Control) was determined with an MDCK plaque assay at 48 hpi. Data are presented as the mean±s.e.m. from three independent experiments. *P=0.014 (D) and P=0.012 (E) versus control as calculated by Student’s t-tests. (F) MDCK cells expressing CFP-RAPEL or CFP (Control) were infected with PR8 at an MOI of 1 PFU per cell for indicated time periods and then subjected to an immunofluorescence-based virus infection assay. Total fluorescence intensities within cells were quantitated and plotted. Data are presented as the mean±s.e.m. (n≥15 from three independent experiments). *P<0.006 versus control as calculated by Welch’s t-test with Bonferroni correction. (G) MDCK cells expressing CFP-RAPEL or CFP (Control) were infected with PR8 at an MOI of 10 PFU per cell for 1 hour. The cells were then subjected to immunofluorescence-based internalization assay as described in the Materials and Methods. Rab7- and NP-positive regions were extracted and their colocalization was determined. Data are means±s.e.m. (n≥20). *P=0.0035 versus control cells, as cal [file csf_44_19001_2.pdf]

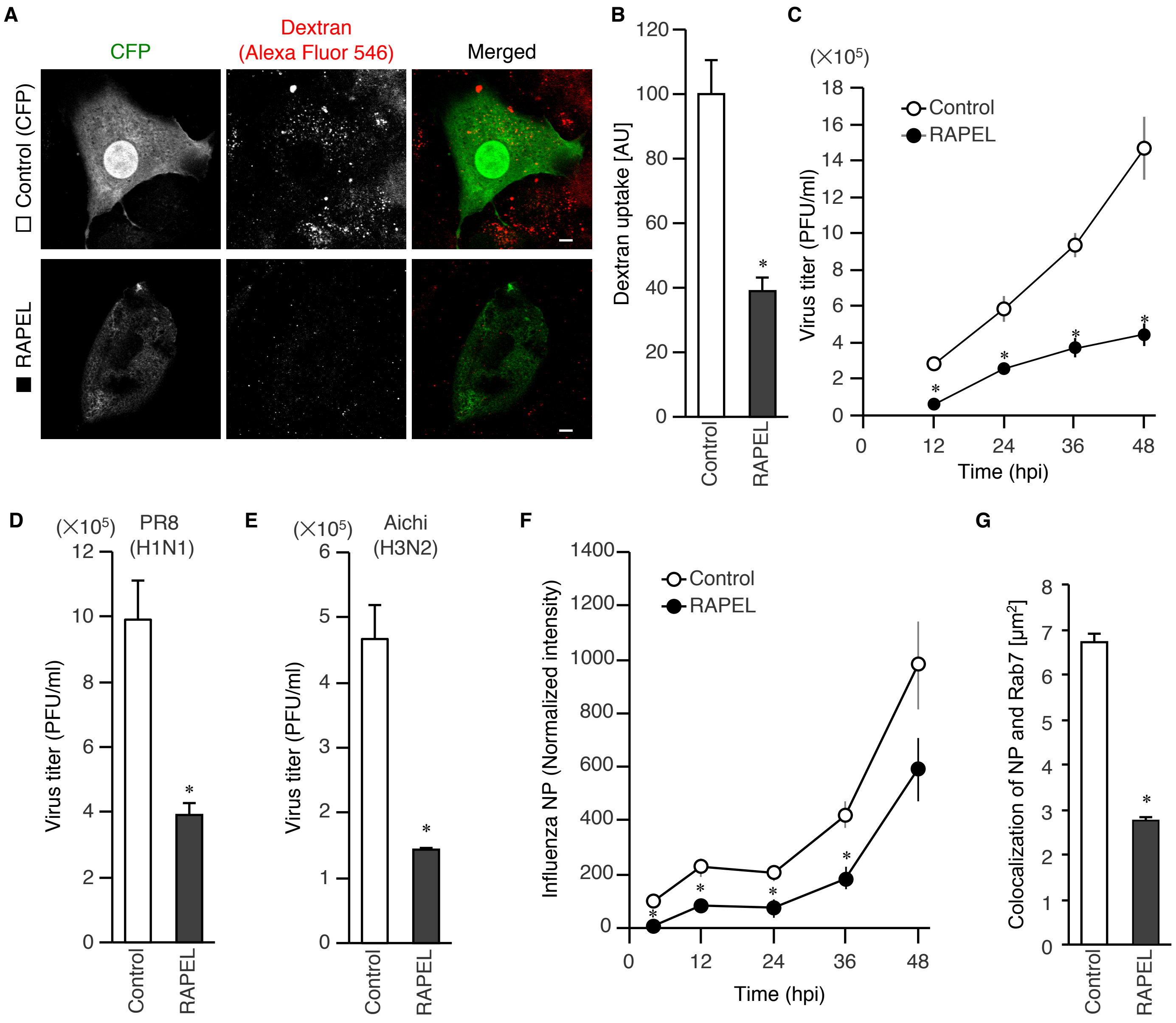


Fig. S2 Fujioka, Satoh et al.

**Fig. S2. RAPEL expression inhibited endocytosis, IAV infection, and internalization.** (**A**) MDCK cells were transfected with expression vectors for CFP (Control) or CFP-RAPEL. After 24 hours, the cells were incubated with rhodamine B-conjugated dextran (70-kD) for 10 min at 37°C and then washed with acidic buffer and PBS. Representative images are shown (**A**). Bar, 10 μm. Total fluorescence intensity within cells was quantitated and plotted (**B**). Data are presented as the mean ± s.e.m. ($n \geq 30$ from three independent experiments). *$P <$ 0.0001 versus control as calculated by Student's *t*-tests. (**C**) 293T cells expressing CFP-RAPEL or CFP (Control) were infected with PR8 at an MOI of 1 PFU per cell for 1 hour. The titer of infectious virus particles released into culture medium was determined with an MDCK plaque assay at 12, 24, 36, and 48 hours post infection (hpi). (**D**, **E**) Replication of PR8 (**D**) or Aichi (**E**) in 293T cells expressing CFP-RAPEL or CFP (Control) was determined with an MDCK plaque assay at 48 hpi. Data are presented as the mean ± s.e.m. from three independent experiments. *$P = 0.014$ (**D**) and $P = 0.012$ (**E**) versus control as calculated by Student's *t*-tests. (**F**) MDCK cells expressing CFP-RAPEL or CFP (Control) were infected with PR8 at an MOI of 1 PFU per cell for indicated time periods and then subjected to an immunofluorescence-based virus infection assay. Total fluorescence intensities within cells were quantitated and plotted. Data are presented as the mean ± s.e.m. ($n \geq 15$ from three independent experiments). *$P < 0.006$ versus control as calculated by Welch's *t*-test with Bonferroni correction. (**G**) MDCK cells expressing CFP-RAPEL or CFP (Control) were infected with PR8 at an MOI of 10 PFU per cell for 1 hour. The cells were then subjected to immunofluorescence-based internalization assay as described in the Materials and Methods. Rab7- and NP-positive regions were extracted and their colocalization was determined. Data are means ± s.e.m. (n ≥ 20). *$P = 0.0035$ versus control cells, as calculated by Student's *t*-test.
